# Diffuse large B cell lymphoma in rheumatoid arthritis express polyreactive and autoreactive BCRs binding protein modifications

**DOI:** 10.1186/s13075-026-03937-0

**Published:** 2026-10-27

**Authors:** Nora Euler, Ana Cristina Gonzalez-Sanchez, Elizabeth Clark, Hedvig Mildner, Marina Galesic, Andrea Kemp, Mattias Jangard, Erik Hellbacher, Nils Landegren, Lina Marcela Diaz-Gallo, Lars Klareskog, Vivianne Malmström, Eva Baecklund, Caroline Grönwall

**Affiliations:** 1 Division of Rheumatology, Department of Medicine Solna, Center for Molecular Medicine, Karolinska Institutet, Karolinska University Hospital, L8:04, Visionsgatan 18, Stockholm, 17176 Sweden; 2 Science for Life Laboratory, Department of Medical Biochemistry and Microbiology, Uppsala University, Uppsala, Sweden; 3 Department of Otorhinolaryngology, Sophiahemmet Hospital, Stockholm, Sweden; 4 Department of Medical Sciences, Rheumatology Uppsala University, Uppsala, Sweden

**Keywords:** Rheumatoid arthritis, Diffuse large B-cell lymphoma, Anti-malondialdehyde acetaldehyde, BCR sequencing

## Abstract

**Background:**

An important risk factor for diffuse large B-cell lymphoma (DLBCL) development in patients with rheumatoid arthritis (RA) is high cumulated disease activity. B cell intrinsic factors and autoimmune processes may contribute, and we hypothesize that BCR autoreactivity may drive lymphomagenesis and/or lymphoma cell survival. RA is primarily associated with anti-citrullinated protein autoantibodies but patients can also have other anti-modified protein autoantibodies, e.g. towards malondialdehyde acetaldehyde (MDA/MAA) adducts which correlate with disease activity and inflammation. Here, we investigated DLBCL properties from RA patients with concurrent lymphoma by B-cell receptor (BCR) next generation sequencing and re-expression.

**Methods:**

In total 22 RA-DLBCL patients were included from the Swedish AUTO-LYMPHOMA cohort. Tumour DNA was extracted from fixed tissues and full length VH/VL fragments were amplified by multiplex PCR and sequenced using Illumina. Computational analysis assessed clonal variations. Dominating sequences were synthesized and expressed as recombinant monoclonal antibodies for antigen binding analysis.

**Results:**

Complete VH/VL lymphoma tissue sequences were generated from nine RA-DLBCL patients. Clonotype diversity within the tumour was generally low and the dominant paired heavy and light chain BCR sequences were obtained for six patients. For three patients, amino acid variations in the DLBCL clonotype indicated intra-clonal evolution. Two of six patients had tumour BCRs with few VH and VL somatic hypermutations (≤ 5 mismatches) while three had high mutations (> 15 VH or VL mismatches). No bias in VH/VL usage was detected. Six lymphoma BCRs from five patients were expressed as recombinant monoclonal antibodies and their binding to autoantigens was assessed by ELISA and a solid-phase microarray (> 400 antigens). All patient-derived lymphoma BCRs were CCP2 negative, but five bound MDA/MAA-protein. Three of these displayed polyreactive binding by in-house assays of which two further displayed strikingly broad polyreactivity on the antigen array. Importantly, for one patient, the lymphoma BCR had high specificity to MDA/MAA-protein, and another patient DLBCL-BCR had binding only to methylated protein.

**Conclusions:**

The data suggest a possible link between polyreactivity and anti-MDA/MAA autoreactivity in RA-DLBCL lymphomagenesis. We hypothesize that high inflammation burden could increase antigen load which may trigger polyreactive and modified-protein B cell responses and/or maintain lymphoma cell survival.

**Supplementary Information:**

The online version contains supplementary material available at https://doi.org/10.1186/s13075-026-03937-0.

## Introduction

 Several autoimmune diseases are associated with an increased risk of developing B-cell lymphoma including Sjögren’s disease, systemic lupus erythematosus and rheumatoid arthritis (RA) [1]. RA patients have been described to have a 2–3 fold increased risk of B-cell lymphoma compared to the general population [2–4], with high disease activity being considered the strongest risk factors [4]. The importance of inflammation is further highlighted in a recent study by Hellgren et al. which showed that the introduction of effective biological disease-modifying antirheumatic drugs (bDMARDs) may have reduced lymphoma risk in RA patients [5]. The majority of RA patients with lymphoma have diffuse large B-cell lymphoma (DLBCL) [6]. Based on the tissue gene expression profile, DLBCL can be subdivided into the subtypes germinal centre B cell-like (GCB), unclassified non-GCB or activated B cell-like (ABC) [7–9]. DLBCL in RA patients (RA-DLBCL) are often of an ABC or non-GCB subtype [10]. ABC DLBCL cell lines have been shown to be reliant on tonic BCR signalling for their survival [11, 12].

Hence, it has been hypothesised that certain DLBCL BCR, also in non-autoimmune individuals, bind to self-antigens, which would drive a chronic BCR activation that sustain survival of the lymphoma cells. Notably, this has only been validated in cell line studies [13]. Importantly, in autoantibody-positive patients, it is unclear to which extent autoantigen-driven BCR activation is critical for lymphoma development and, if so, what the specificity of these BCRs is, and if it is related to the underlying autoimmune disease. Answering these questions is of high interest not only for RA-associated lymphomas, but also for other autoimmune-disease associated lymphomas where an increased understanding of mechanisms and specificities involved in lymphoma development may help both to predict the risk for lymphomas and to identify new and specific targets for therapy.

In RA, a majority of patients have anti-citrullinated protein antibodies (ACPA) and/or rheumatoid factor (RF) antibodies [14]. RA patients have also been shown to have elevated IgG antibodies to malondialdehyde acetaldehyde (MAA) modified proteins. While anti-MAA antibodies are not RA specific, they correlate with RA disease activity and markers of inflammation (e.g. DAS28-ESR, IL6, TNF) [15–17]. Additionally, RA patients have been shown to exhibit significant shifts in B cell populations with B cells showing an increased expression of activation-associated markers such as HLA-DR and CD11c and expansion of CD27^−^ class-switched cells and CD21^low^ cells [18, 19].We have previously reported high seropositivity among RA-DLBCL patients and higher levels of plasma biomarkers associated with B cell activation compared to RA patients without lymphoma [20].

In the present study we analyzed the intra-clonal evolution and autoantigen-specificity of BCRs cloned from DLBCL of RA patients. We took advantage of the unique Swedish national AUTO-LYMPHOMA cohort with RA patients that have developed DLBCL and sequenced the immunoglobulin V(D)J domain of the BCR in formalin-fixed paraffin embedded (FFPE) tumour tissues. The dominant tumour sequences were identified and were expressed as recombinant monoclonal antibodies that were tested for their binding to autoantigens.

## Materials and methods

### Study population

In total 22 RA patients with DLBCL (45% male, median age of 70 years) from the Swedish AUTO-LYMPHOMA cohort [20] were included for BCR tumour tissue sequencing and Epstein-Barr-virus EBV testing by qPCR (Supplemental Fig. 1). DLBCL tissues from nine RA-DLBCL patient generated DNA of high enough quality for BCR sequencing (Supplemental Tables 1 and 2, Supplemental Fig. 1). The RA-DLBCL patients were diagnosed with lymphoma between the year 2010 to 2021 in accordance with the World Health Organization classification of Tumours of Haematopoietic and Lymphoid Tissues [21]. All RA-DLBCL patients fulfilled the 2010 ACR/EULAR RA classification criteria [22]. All patients gave informed consent to participate in the study, and the study followed the Helsinki declaration and was approved by the regional ethics review boards.

### BCR library preparation and sequencing

For BCR sequencing FFPE tumour tissue samples were sectioned into four 10 µm thick slices and transferred into a sterile 1.5 mL microtube by the responsible biobank. DNA was extracted using the AllPrep DNA/RNA FFPE kit (QIAGEN). A tonsil sample from a healthy donor was fixed with 4% paraformaldehyde, paraffin embedded and used as a control (Histocore facility at Karolinska Institutet). The concentration and quality of nucleic acid extractions were determined by Agilent Tapestation 2000 using Genomic DNA Screen Tape and thereafter stored in -80°C until further processing. FFPE DNA was used to prepare a BCR library for the V(D)J domain using an in-house multiplex PCR protocol. The heavy chain and the kappa and lambda light chain were amplified separately using forward primers targeting the variable chain and a reverse primer targeting the J-chain (Supplemental Table 3). Template amount and conditions were optimized using FFPE DNA from a healthy donor tonsil sample (Supplemental Fig. 2). FFPE DNA tumour template input 1 ng – 67.8 ng was used for each PCR reaction (Supplemental Table 4). The following thermal cycling program was used for PCRI: 95°C for 15 min, 45 cycles of 94°C for 30 sec, and 54°C for 30 sec, 72°C for 1 min, followed by 72°C for 10 min. For the kappa and lambda light chain PCRI products, an additional PCR was performed using primers that target the overhang in both the 5’ and 3’ end (introduced in the first PCR). The following thermal cycling program was used for light chain PCRII: 95 °C for 15 min, 8 cycles of 94 °C for 30 s, 58 °C for 30 s, 72 °C for 1 min, followed by 72 °C for 10 min. The PCR heavy chain and PCR light chain products were thereafter purified using MinElute gel purification (QIAGEN). Illumina adapters with Nextera DNA indexes were added by PCR to the 5’ and 3’ to generate individual libraries with a unique combinatorial index (Supplemental Table 5). The following thermal cycling program was used for PCR: 95 °C for 15 min, 13 cycles of 94 °C for 30 s, and 58 °C for 30 s, 72 °C for 1 min, purification kit by (QIAGEN). For all PCR reactions, Hotstar Taq DNA polymerase and the accompanied PCR buffer provided were used according to the instructions by the manufacturer. The base pair length of each individual library validated using Agilent Tapestation 2000 (D1000 Screen Tape) and the concentration determined using Qubit dsDNA Broad Range Assay (Thermo Fisher Scientific). The individual libraries were pooled and sequenced using Illumina (Nextseq 2000, 2 × 300 bp) at National Genomics Infrastructure (NGI) at Science for Life Laboratories (Stockholm, Sweden).

### EBV quantitative PCR

We determined the Epstein Barr virus (EBV) infection status for all the tumour tissues included by qPCR. 1–2 µl DNA template (5 pg-50 ng) were used in one 20 µl reaction with IQ SYBR Green Supermix (Bio-Rad) and primers. Previously reported EBER2 specific primers (Fwrd: AAC GCT CAG TGC GGT GCT A; Rev: GAA TCC TGA CTT GCA AAT GCT CTA) were used to detect EBV (resulting in a 90 bp amplicon) [23]. In parallel, a 108 bp amplicon of β-actin was detected as housekeeping control (primers: Fwrd: TCG TGC GTG ACA TTA AGG AG; Rev: CAG GCA GCT CGT AGC TCT TC). The following thermal cycling program was used: 95 °C for 3 min, 40 cycles of 95 °C for 10 s, and 60 °C for 20s, followed by 55 °C to 95 °C in 0.5 °C increments for 20 s, on a Bio-Rad CFX384 Real-Time system with the C1000 Touch Thermal cycler (Bio-Rad). The EBV- B cell line LP-1 (DSMZ ACC41) was used as negative control and the EBV^+^ B cell line Raji (DSMZ ACC319) was used as positive control. Relative expression was calculated using the delta-delta Ct method, where samples were normalized to β-actin, and LP-1 was used as the relative control group. If Cq value could not be determined, a Cq value of 40 was assigned for calculation purposes.

### Bioinformatic analysis

The nf-core/airflow (V.4.2) bioinformatic pipeline was used for analysis of the BCR sequences [24]. In the first quality control and sequence assembly the forward and reverse primer sequences were masked and sequences were filtered for the removal of adapters/primer sequences, and low-quality bases/reads with a phred score of < Q30 and read size < 250 bp. The number of reads before and after sequence assembly and quality filtering can be seen in Supplemental Table 6. Sequences were annotated by IgBlast [25] using the IMGT germline reference (Change-O AssignGenes). Non-productive and VDJ sequences with junction lengths not divisible by three were removed. A decontamination step was applied to mitigate cross-sample carryover. Sequences shared across samples with identical V gene, J gene, and CDR3 were considered potential carryover and were retained only in the sample with the highest sequence count and removed from other samples. Clonotype was defined by the same VH gene and JH gene and by nucleotide junction (CDR3) similarity. The clonal assignment threshold was determined by calculating the nearest neighbor distances using length-normalized nucleotide Hamming distance distances to identify the optimal cutoff separating clonally related from unrelated sequences (Shazam, R package) [26]. The resulting clonal threshold was then used to cluster and assign clonotypes based on junction region nucleotide sequence similarity, with the criteria that sequences within a clonotype share the same variable heavy gene (VH-gene) and heavy J-chain gene (JH-gene) and junction length [27]. For the light chain sequences clonality was investigated by grouping light chain sequences that shared light chain variable region (VL-gene) and J region (JL-gene) gene usage and junction length.

### Monoclonal antibody production

Identified dominant clonotypes from the BCR sequencing were expressed as recombinant IgG monoclonal antibodies as previously described [28]. The variable heavy chain (VH) and light chain (VL) fragments were synthesized and subcloned into human gamma1 and kappa/lambda expression vectors (Integrated DNA Technologies). Heavy and light chain vectors were co-transfected into Expi293 HEK cells (Thermo Fisher Scientific) for IgG1 expression. IgG were purified by protein G affinity chromatography (Cytiva) and eluted fractions were buffer exchanged to PBS using Zeba Spin desalting columns (Thermo Fisher Scientific). Concentrations were estimated by spectrophotometer measurement at 280 nm. Purity and quality of proteins were assessed by SDS-PAGE analysis under reducing and non-reducing conditions on Bolt 4–12% bis-tris gel with MES-SDS running buffer and SimplyBlue SafeStain Coomassie staining (Thermo Fisher Scientific). We expressed and purified control recombinant IgG1 clones with the same system. Negative controls 1362:01E02, 1276:01G09 were derived from synovial memory cells [29, 30]. The ACPA clone 1325:01B09 was derived from a synovial plasma cell [31]. The plasmids for the negative control clone MGO53 and the polyreactive clones JB40 and ED38 were a generous gift from Hedda Wardemann and Eric Meffre and the clones were derived from healthy individuals [30]. The MDA-specific control clones 1276:01F04 and 146+:01G07 were derived from B cells in RA patient synovial fluid or tissue, respectively, as previously reported [15]. Additional control clones used in polyreactive assays are presented in the Supplemental Material.

### Tumour monoclonal recombinant antibody binding and reactivity

Autoreactivity of DLBCL-derived monoclonals was first assessed by a panel of in-house ELISAs. The level of polyreactivity was investigated by coating ELISA plates with a HEK293 soluble membrane protein fraction as previously described [32]. As a reference a standard curve for reactivity was set based on the previously described polyreactive clone JB40 [30]. Based on previous screenings of control monoclonal antibodies, a reactivity over 50 units (U)/ml, which is equivalent to an absorbance (450 nm) around 0.5, is considered to show suspected polyreactivity that merits further testing [32]. Rheumatoid factor activity was assessed by coating ELISA plates with polyclonal rabbit IgG (Jackson ImmunoResearch) at 5 µg/ml and the binding of lymphoma antibodies were investigated at 9 µg/ml in 1% bovine serum albumin (BSA) in PBS. The binding of the lymphoma antibodies to oxidation-associated post-translational modifications were assessed in ELISA by coating wells with unmodified BSA (New England Biolabs) or BSA modified by malondialdehyde (MDA-BSA; Bethyl), carbamylation (Carb-BSA; Academy Bio-Medical Co.), methylation (Met-BSA; Sigma Aldrich), advanced glycan end-products (AGE-BSA; Abcam), phosphorylcholine conjugation (PC-BSA, Biosearch Technologies) or MDA acetaldehyde (MAA-BSA) modified BSA using and in-house protocol as previously described [16, 33]. We also evaluated the binding to low-density lipoprotein (LDL, Academy Bio-Medical Co.), MDA-LDL (Nordic Biosite), nitrate-modified LDL (Nitro-LDL; Nordic Biosite) and oxidized LDL (Ox-LDL; Nordic Biosite) and the phosphorylcholine containing antigen, pneumococcal cell wall polysaccharides (CWPS; Statens serum Institut). We further investigated binding to MAA and citrulline (cit) modified human serum albumin (HSA) generated as previously described [33, 34]. MDA-HSA without MAA residues were generated by addition of the reducing agent sodium cyanoborohydride (SCBH) in the MDA modification protocol as previously described [33]. All antigens were coated at 3 µg/ml. The plates were blocked with 3% BSA in PBS and the lymphoma antibodies were evaluated at a starting concentration of 5 µg/mL, followed by a 2-fold dilution to in 1% BSA in PBS. In all in-house assays, the binding was detected by goat anti-human IgG Fc-HRP (Jackson ImmunoResearch) and TMB substrate (Biolegend). The CCP2 IgG reactivity was tested using the commercial CCPlus IMMUNOSCAN ELISA (SVAR Life Science). Anti-nuclear antibody (ANA) reactivity was assessed by immunofluorescence binding to NOVA Lite HEp-2 ANA slides (Werfen) and visualized by ECHO Revolve fluorescent microscope. The mAbs for the ANA staining were tested at 10 µg/mL following the manufacturer’s instructions. In addition, the binding of the mAbs was also evaluated using a large custom-designed solid-phase microarray including more than 450 full-length human proteins, as previously described [35]. Microarrays were blocked for 90 min at room temperature in blocking buffer (2% bovine serum albumin and 0.05% Tween-20 in PBS), followed by overnight incubation (≥ 16 h) with the mAbs in the same buffer at 5 µg/mL. Arrays were washed five times in PBS with 0.05% Tween-20 and incubated for 90 min with Alexa Fluor 647–conjugated goat anti-human IgG (1:2000, Thermo Fisher Scientific). After additional washing, slides were rinsed in deionized water, dried by tabletop microarray centrifugation, and scanned within 1 h using a GenePix 4000B fluorescence scanner (Axon Instruments) the default settings of GenePix Pro V.5.1.0.19 or GenePix Pro V.7 software with a custom GenePix Array List (GAL) file, which extracts the median fluorescence intensity after subtraction of local background intensities. For each antigen, a mAb signal above the average intensity for six control serums from healthy blood donors at 1:2000 dilution was considered a positive signal.

### BCR statistical and clonal analysis

For the BCR sequencing data analysis, variable gene (V-gene) usage per patient sample was quantified (dplyr, R package). Sequences were grouped by V-gene, and the number of sequences assigned to each V-gene was counted and the relative V-gene frequencies were calculated as the proportion of sequences assigned to each V-gene out of the total number of sequences for the heavy chain and light chain loci, respectively. Clonotype frequency was calculated by the sum of duplicate counts for each clonotype ID, divided by the total duplicate count for the heavy chain loci in the patient sample. The calculated clonotype frequencies for each patient sample were visualized by a pie chart. Clonotypes with a relative frequency < 1% were labeled as ‘other’. The location of amino acid replacements (FW; framework region, CDR1; complementary determining region 1, CDR2; complementary determining region 2) for the identified dominant tumour clonotype was investigated. To do this, the 100 most abundant unique amino acid sequences by sequence reads (proportion of duplicate counts) within the identified tumour clonotype from each patient sample were aligned to the corresponding germline reference sequence, and a mismatch matrix was constructed. For each amino acid position, the number of sequences differing from the germline residue was then calculated. Investigation of intra-clonal diversity was performed by analysis of the amino acid VDJ variability within the clonotype lineage of the dominating tumour sequence.

## Results

### Clinical characteristics

In total 22 RA-DLBCL patients were included in our study from the National AUTO-LYMPHOMA cohort. Among the 22 RA-DLBCL patients, we detected EBV by qPCR in the tumour tissue for three patients (14%) (Supplemental Fig. 3). Tumour tissues from nine RA-DLBCL patients (40% of patients included) were of sufficient quality for PCR amplification and generated high-quality libraries for heavy chain and/or light chains and were subjected to BCR sequencing (Supplemental Fig. 1, Supplemental Table 1). The median age of lymphoma diagnosis for the nine RA-DLBCL patients included for BCR sequencing was 72 years of age (44% were female). Two patients (RAL10 and RAL04) with tumour BCR sequencing, had detectable levels of EBV. Importantly, for both these patients we had access to multiple tissue samples. For RAL10, all investigated biopsies were EBV positive, while for RAL04 only one out of two had detectable levels of EBV. Due to tumour DNA quality, we could only generate BCR libraries from the RAL04 tissue that did not have detectable EBV (Supplemental Fig. 3). Among the nine RA-DLBCL patients included for BCR sequencing, seven patients (78%) had anti-CCP2 IgG and/or RF IgM serum positivity. Notably, six of the nine RA-DLBCL patients included for tumour BCR sequencing had initiated anti-CD20 rituximab therapy as part of their lymphoma treatment before blood sampling, which may affect the antibody levels. All RA-DLBCL patients had established RA disease (> 2 years disease duration, median 21 years duration) and 89% had received disease modifying anti-rheumatic drugs (DMARDs) for their RA before lymphoma diagnosis (Supplemental Table 2).

### Immunoglobulin V(D)J gene usage

**Fig. 1:**
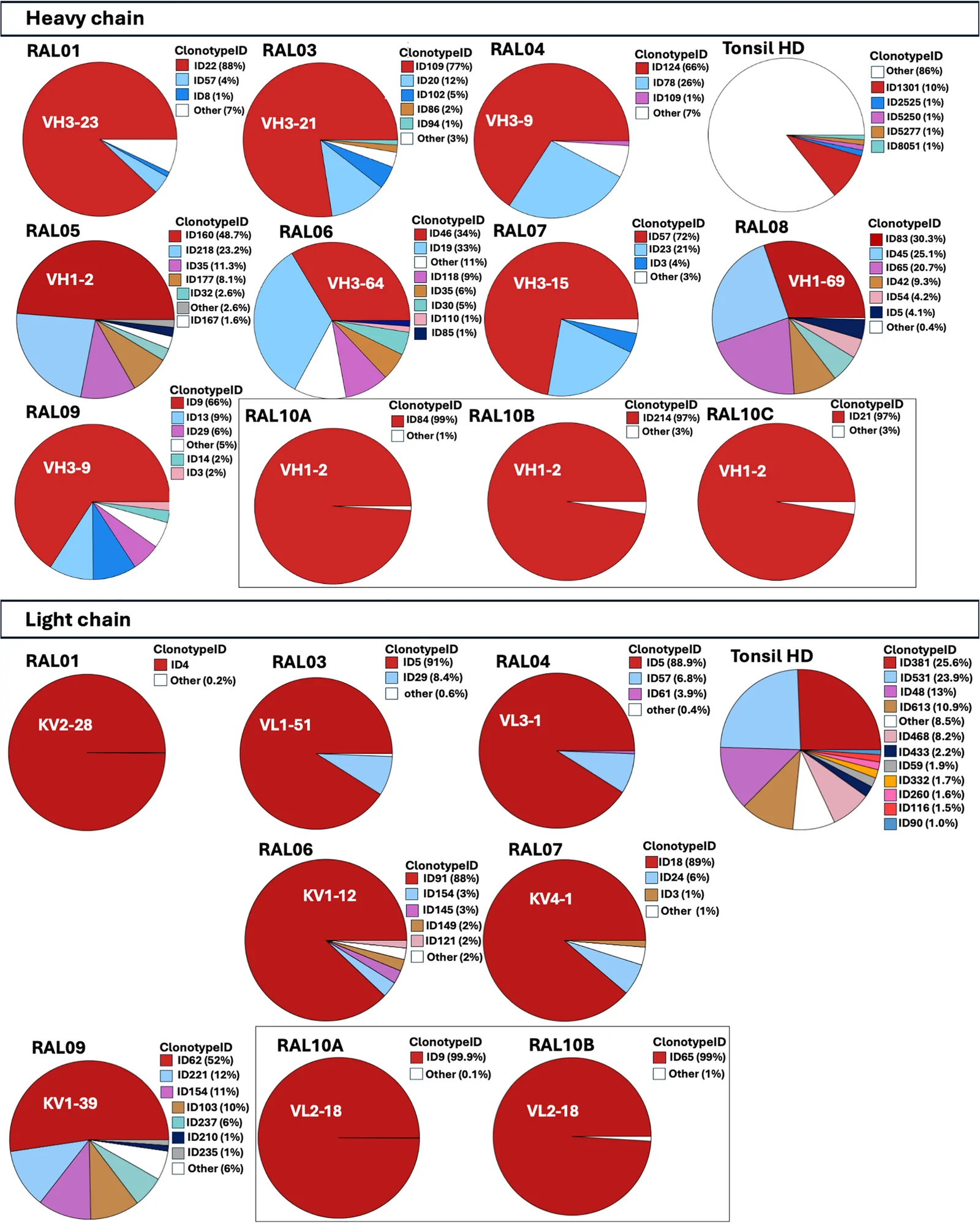
Expanded heavy chain and light chain clonotypes in tumour tissue from RA-DLBCL patients. BCR sequences were obtained by Illumina sequencing of DNA from 9 formalin-fixed, paraffin-embedded, lymphoma tissues from RA patients with concurrent diffuse large B-cell lymphoma (RAL01, RAL03, RAL04, RAL05, RAL06, RAL07, RAL08 and RAL10). Tumour sequence variability was investigated by clonotype analysis. For the heavy chain the clonotype was defined by the same VH gene and JH gene and by nucleotide junction (CDR3) similarity (heavy chain variable regions). For the light chain sequencing data, the clonality was defined by the same VL gene and JL gene and by the same junction length. For both the VH and VL data the frequency of each clonotype within all obtained sequences was calculated for each patient sample and visualized as a pie chart. Clonotypes which had the relative frequency <1% were labeled as “other” (white). Tonsil from a healthy donor (HD) was used as control. Three independent biopsies (RAL10A, B, C) were investigated for patient RAL10

We sequenced tumour BCR from RA-DLBCL patients to investigate the tumour BCR characteristics (e.g. SHM, N-glycosylation sites and variable gene usage). Full-length recombined VH and/or VL regions were amplified with multiplex PCR from formalin-fixed paraffin-embedded tissue sections from RA-DLBCL patients and subjected to Illumina sequencing. VH PCR and sequencing were successful for nine patients, and VL sequencing was successful for tumour tissues from seven patients. For one patient (RAL10) we sequenced three different biopsies (Supplemental Fig. 1). Raw sequences (Supplemental Table 6) were carefully quality control filtered and annotated for gene usage and clonotype (VH-JH recombination and HCDR3 length and similarity or VL-JL recombination and LCDR3 length) to determine tumour heavy chain and light chain clonotypes (Fig. 1, Supplemental Tables 7–9). The final number of annotated complete V(D)J sequences varied between the analyzed tissues (Supplemental Tables 8–9). For six RA-DLBCL patients a dominant VH clonotype could be identified that corresponded to 66–99% of the patient heavy chain sequencing data (Table 1; Fig. 1, Supplemental Table 7). Within the dominating clonotype lineages, the most frequent unique sequence represented 14–71% of the total reads in the clonotype (Supplemental Tables 8–9, Supplemental Fig. 4–5). In sequence alignments, single nucleotide variations with < 5% frequencies among the reads in the clonotype are likely originating from PCR or sequencing errors while more frequent variants may instead reflect intra-clonal variations (visualized in Supplemental Fig. 4–5). Hence, the dominating unique sequence could be identified with high confidence in the data.

**Table 1:** An overview of identified lymphoma BCR sequences from RA-DLBCL patients selected to be expressed as recombinant monoclonal antibodies

Patient	Expressed mAb	V	J	SHM	*N*_glyc_	CDR3
RAL01	RAL01:C22	VH3-23	JH4	13	1	CAKAQGFGRSWPFDYW
	-	VK2-28	JK4	28	1	DIVMTQSP
RAL03	RAL03:C109	VH3-21	JH6	19	0	CARDLIRFGGFIRGMDVW
	-	VL1-51	JL3	19	0	CATWDRSLSGGVF
RAL04	RAL04:C124	VH3-9	JH6	4	0	CAKESTVTTNGMDVW
	-	VL3-1	JL2	1	0	CQAWDSSTVVF
RAL09	RAL09:C9	VH3-9	JH4	9	0	CTIDRGSYSNEAYYYGMDVW
	-	VK1-39	JK4	1	0	CQQSYSTLTF
RAL10^#^	RAL10:C214	VH1-2	JH4	3	0	CAKAQGFGRSWPFDYW
	-	VL2-18	JL2	3	1	CSLYTSSSTFNVVF
RAL10^#^	RAL10:C84	VH1-2	JH4	4	0	CAKAQGFGRSWPFDYW
	-	VL2-18	JL2	3	1	CSLYTSSSTFNVVF

#Two monoclonals were generated from patient RAL10 with identical VL but one amino acid different in VH

Two of six patients had tumour BCRs with low level of variable heavy chain (VH) and variable light chain (VL) somatic hypermutations (SHMs) in the dominating sequence (≤5 mismatches compared to the closest IMGT germline allele) and three patients had high number of SHMs (> 15 mismatches in VH or VL). Among the six RA-DLBCL patients with identified lymphoma BCRs, two patients had a SHM-introduced N-glycosylation sites in the VH and/or VL sequence. For five of the patients the lymphoma VH gene belonged to the VH3 family, and one patient (RAL10) had a dominant VH1-2 gene expression in the tumour tissue.

### Evidence for lymphoma BCR antigen-selection and continued BCR evolution in the tumour

**Fig. 2:**
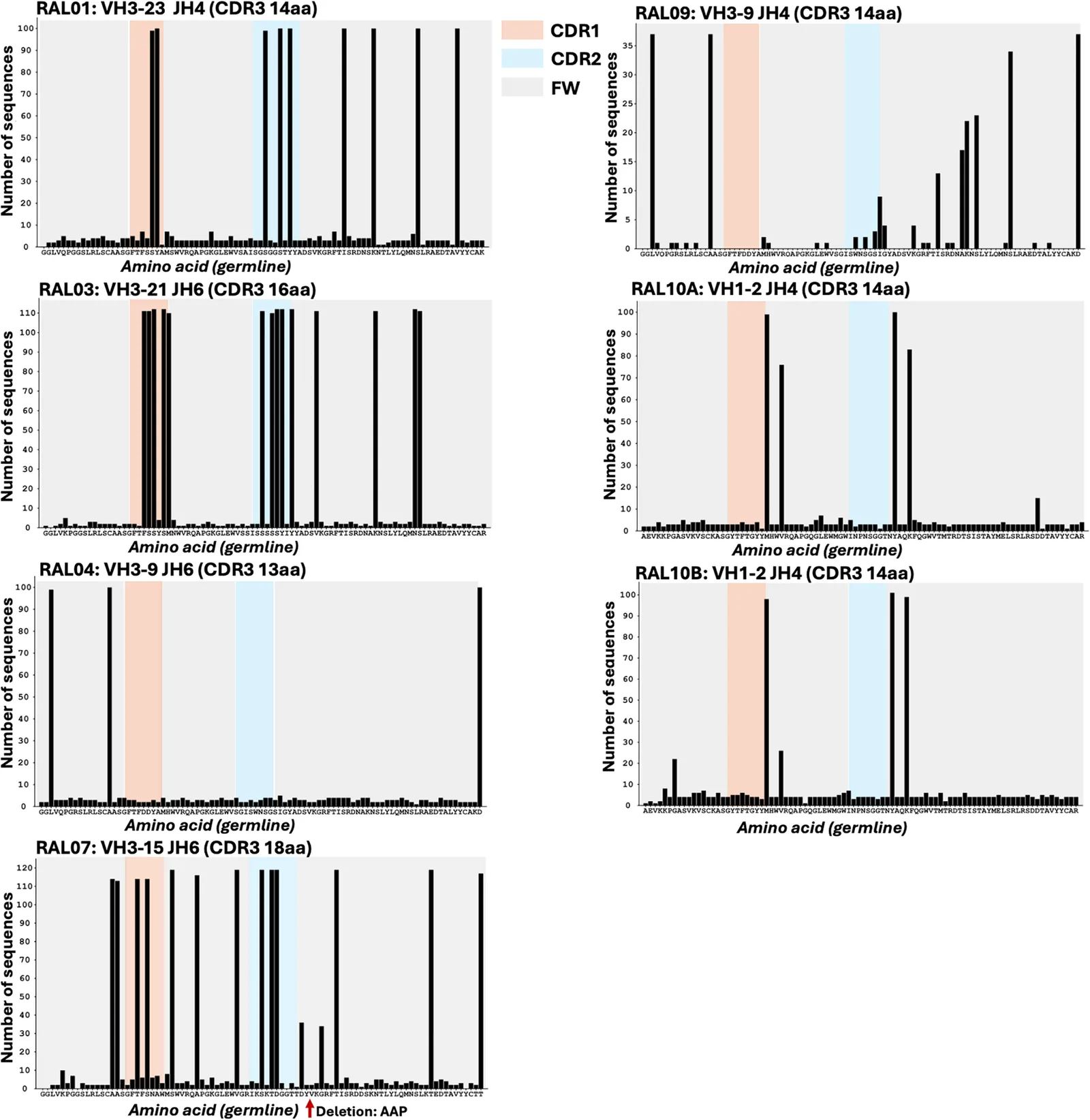
Location of somatic hypermutations in tumour tissue from RA-DLBCL patients. Within the top 100 most frequent sequences in the identified dominating tumour clonotype, we mapped the number of heavy chain sequences with a mismatch compared to the closest VH germline reference in the IMGT database, and their location within the variable region e.g. framework (FW), CDR1, CDR2 by amino acid alignment.The number of sequences are shown on the y-axis and the amino acid sequence is shown on the x-axis with the corresponding CDR1 region in orange, CDR2 in blue, and the FW region in grey. A shift in the sequence due to e.g deletion, is highlighted using a red arrow pointing towards the specific site where the shift is found. Data is shown for VH from six patient from where a dominating clonotype could be identified (RAL01, RAL03, RAL04, RAL07, RAL09 and RAL10). For RAL10 sequences from two different biopsies are presented (RAL10A and RAL10B)

To evaluate whether the observed mutations in the VH region of the identified tumour clonotypes are indicative of antigen-driven selection, we first investigated the location of replacement mutations in the VH gene for the most abundant sequences within the identified tumour clonotype. We found that tumour BCR from patient RAL01, RAL03 and RAL07 had somatic hypermutations that resulted in amino acid replacements in both the complementary determining region (CDR) and framework region (FW) (Fig. 2).

**Fig. 3:**
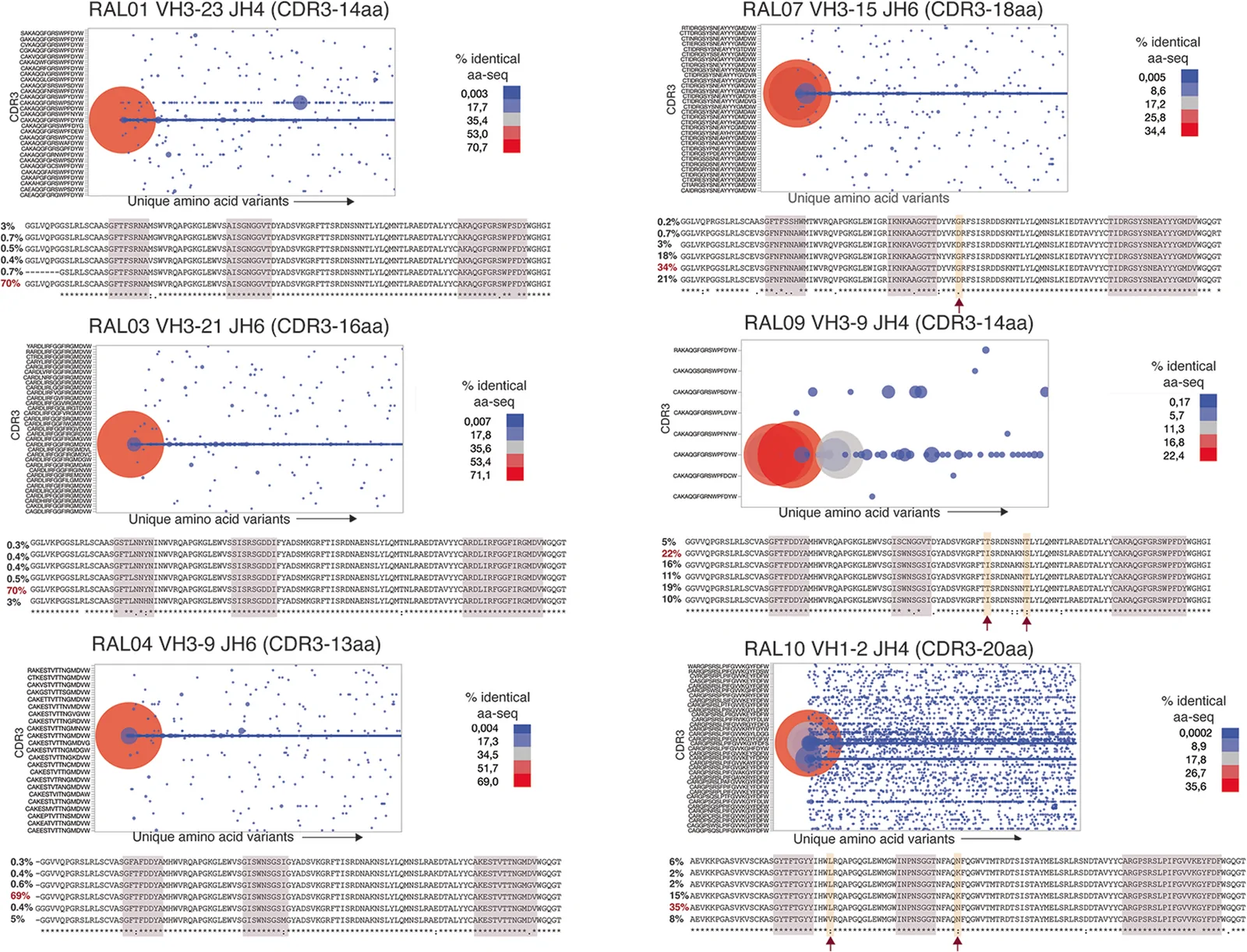
Intra-clonal lymphoma BCR amino acid sequence variability. Intra-clonal evolution was investigated by assessing the amino acid diversity of the full VDJ sequences within the tumour VH clonotype lineages (defined by the same VH-JH and CDR3 length as the dominating sequence). The bubble graphs are visualizing proportions of different unique sequences by full amino acid sequence vs CDR3 sequence. The linages for patient RAL01 (850 sequences of VH3-23 JH4 lineage) RAL03 (547 sequences of VH3-23 JH6 lineage), RAL07 (1045 sequences of VH3-25 JH6 lineage), RAL09 (52 sequences of VH3-9 JH4 lineage) and RAL10 (13329 sequences of VH1-2 JH4 lineage from three different biopsies) are visualized. Larger bubbles in red color indicate a higher proportion of a unique amino acid sequence among the total lineage sequence reads (by number unique nucleotide sequences multiplied by read counts). Alignment of the top six identified tumour amino acid sequences are shown below the bubble graph with indicated proportion of total reads in the lineage. Sites with clear conversion towards different amino acids in multiple independent sequences are highlighted in yellow and with arrows

The deep sequencing also made it possible to investigate continuous intra-clonal evolution of the lymphoma cells. When analyzing all amino acid sequences within the dominating VH lineage (by V-J and CDR3 length), we observed diversity throughout the sequences. We could detect sequence conversions of variants that due to the high frequency are unlikely to be explained by random PCR and sequencing errors. Hence, this provided evidence of intra-clonal evolution. For three patients (RAL01, RAL03 and RAL04) the most prevalent amino acid sequence comprised of 70% of the total sequencing reads within the lineage and the second most prevalent comprised of 3–5%. In contrast, for the other three patients (RAL07, RAL09 and RAL10) we could detect several prevalent variants occurring in 15–35% of sequences within the clonotypes and alignment of the six most prevalent amino acid sequences showed conversion of specific residues. Notably, these sites were located in the framework region so even though they prove that there is an ongoing clonal evolution of the tumour cells it does not necessarily have to be antigen-driven (Fig. 3).

To further evaluate antigen binding by the lymphoma BCR, we expressed the identified dominating heavy and light chain tumour sequences as recombinant monoclonal antibodies for the six patients where it could be identified (Table 1). For patient RAL10 we identified the same dominant tumour sequence for the three different tumour sections. Interestingly, for patient RAL10, we also decided to express two variants of the heavy chain sequence detected in the intra-clonal diversity analysis, which also represented the most abundant unique sequences in two independent biopsies, to investigate if it would affect binding properties. These sequences, RAL10:C84 and RAL10:C214, differed by one nucleotide which altered the amino acid from V37 (germline version) to L37 in framework region 2 (FWR2). One clone showed poor expression levels (RAL:07) and were excluded from further analysis. The remaining six recombinant monoclonal lymphoma antibodies were validated to be of correct size by SDS-PAGE (Supplemental Fig. 6).

### Lymphoma BCRs display predominant polyreactivity and binding to malondialdehyde protein adducts

**Fig. 4:**
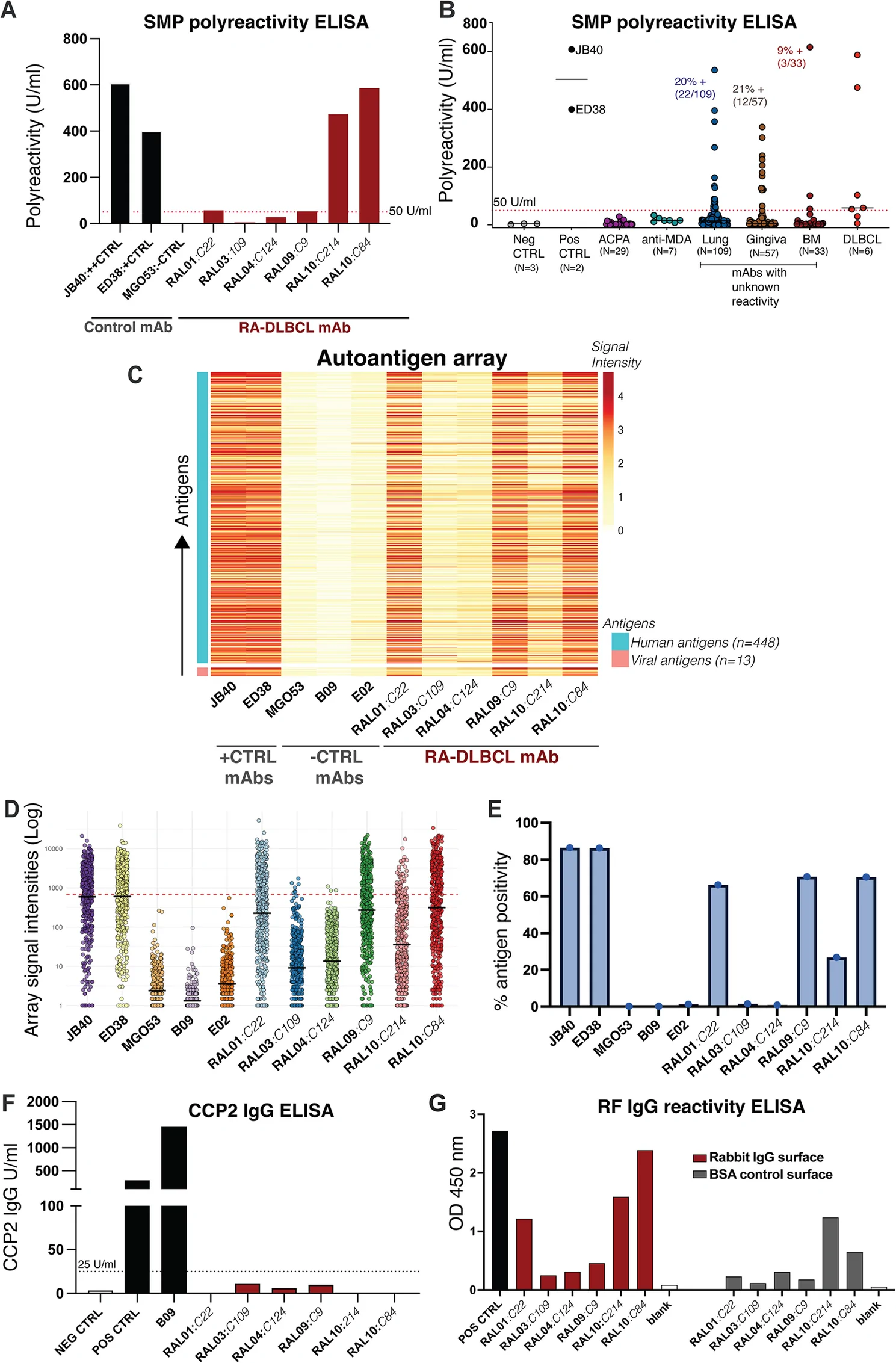
Some lymphoma BCR display polyreactive binding. Six recombinant lymphoma BCR antibodies were produced as hIgG1 from five RA-DLBCL patients (RAL01, RAL03; RAL04, RAL09, RAL10) and evaluated for binding reactivity. Two antibodies with a single amino acid difference were produced from patient RAL10. **A**. All recombinant lymphoma antibodies were tested for polyreactivity at 5 μg/mL in an in-house SMP-polyreactivity ELISA. The human monoclonal antibody MGO53 was used as a negative control and the previously reported polyreactive clones JB40 and ED38 were used as positive controls. **B**. SMP ELISA polyreactivity of the tumour DLBCL antibodies compared to positive and negative control clones as well as previously published RA single cells derived anti-CCP2+ ACPA and anti-MDA/MAA clones as well as clones with unknown reactivity from lung (bronchoalveolar lavage fluid), gingiva tissue and bone marrow plasma cell (see Supplemental Table 10). All human mAbs were expressed recombinantly in the same system and evaluated at 5 μg/ml with values normalized towards a standard curve of JB40. **C**-**E**. The tumour antibodies were also tested in a large antigen array (>400 antigens) in parallel with the negative control human monoclonals MGO53 and E02 (without know reactivity) and the ACPA mAb 1325:01B09 (B09; with no binding to native proteins) and the polyreactive clones JB40 and ED38.C. Heatmap visualizations of the array signal intensities. **D**. Log10-transformed array signals for each monoclonal. The red line is showing the average signal for six healthy control serums diluted 1:2000. **E**. Proportion of positive antigens on the array per antibody, using the average IgG signal for six healthy control serum for each antigen as cutoff for positivity. Panel **D-E** include only human antigens, panel C show values also for viral control antigens. **F**. CCP2 binding of DLBCL mAbs at 5μg/ml IgG1 using SVAR Life Science Immunoscan CCPlus IgG ELISA. The kit provided negative (NEG CTRL) and positive (POS CTRL) controls are shown as well as the ACPA mAbs 1325:01B09 (B09). **G**. RF-reactivity of the DLBCL mAbs was evaluated at 9µg/ml by an in house ELISA comparing coated rabbit IgG to a control surface of bovine serum albumin (BSA). A pool of serum from RF IgG positive RA patients were used as control (POS CTRL, 1:4000 dilution)

The six recombinant monoclonal antibodies produced from lymphoma BCR from five RA-DLBCL patients were investigated for polyreactive binding using an in-house soluble membrane protein (SMP) fraction ELISA developed to assess polyreactivity of monoclonal antibodies [30]. The cutoff for the assay has previously been established by analysis of > 200 RA-derived monoclonal IgG1 antibodies expressed in the same system (Fig. 3) [32, 36, 37]. In the SMP ELISA, the two lymphoma BCRs from patient RAL10 were clearly polyreactive in the same range as well-established polyreactive clones used as controls in the assay [30] and two clones (RAL01 and RAL09) were borderline positive (Fig. 4).

**Fig. 5:**
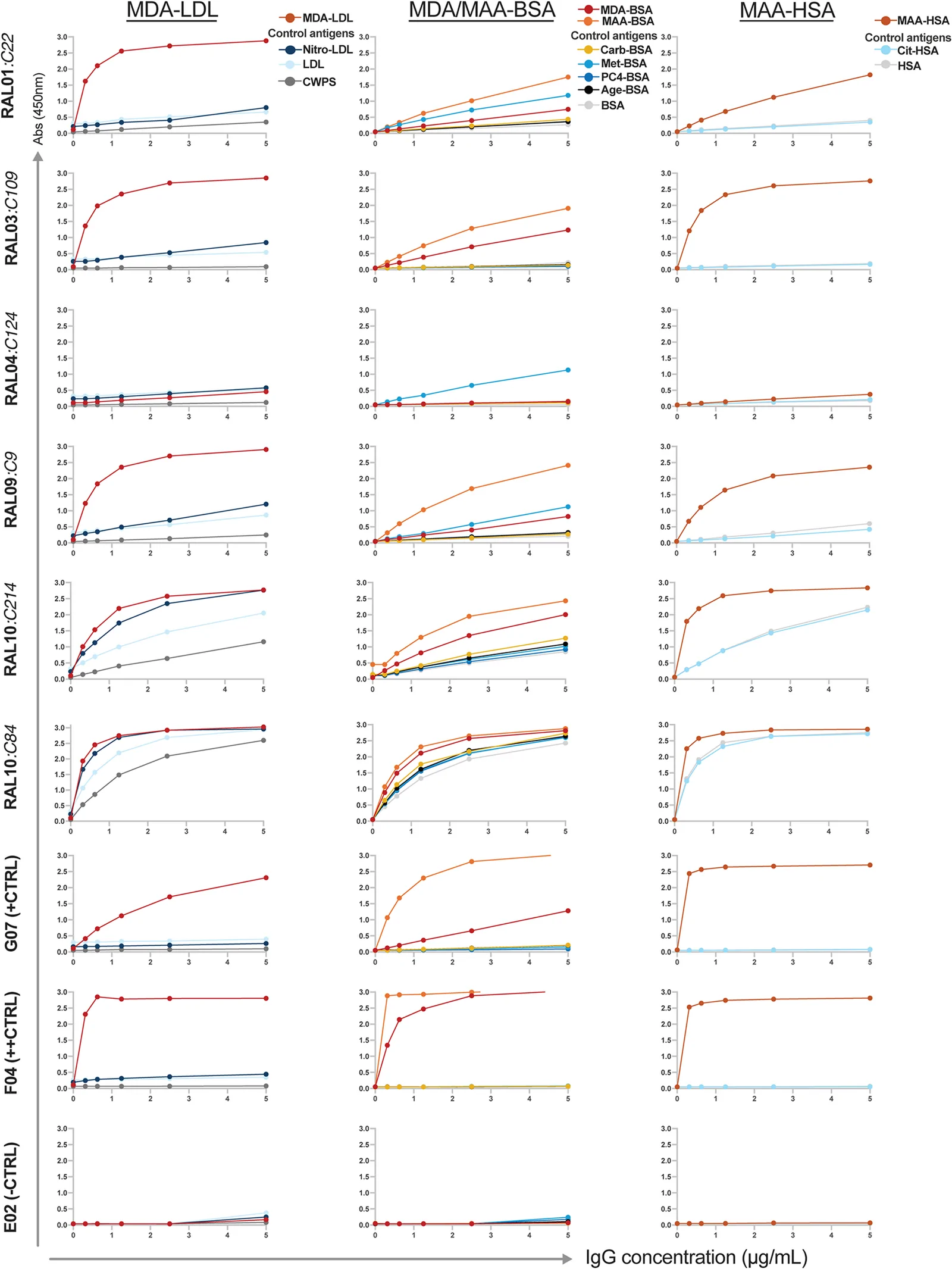
Lymphoma BCR show binding to MDA/MAA modified proteins. The six DLBCL tumour BCR derived mAbs from five RA-DLBCL patients (RAL01, RAL03; RAL04, RAL09, RAL10) were tested for binding to different malondialdehyde (MDA) and malondialdehyde acetaldehyde (MAA) modified antigens and control antigens by in-house ELISA. The recombinant human IgG1 were tested at indicated concentration, starting at 5 μg/mL followed by a two-step serial dilution. In parallel the synovial fluid derived anti-MDA/MAA positive control antibodies 146+:01G07 (G07, MAA-specific) and 1276:01F04 (F04, MDA-specific) [15] and negative control clone 1361:01E02 (E02) were included at the same concentrations. The absorbance was measured at 450 nm. Both MDA and MAA modified proteins can contain different adducts (e.g. linear MDA: N-ε-(2-propenal)-lysine and ring-formed MAA: dihydropyridine (DHP)-lysine and intra-molecular cross-links). LDL: low density lipoprotein; BSA: bovine serum albumin; HSA: human serum albumin; AGE: advanced glycan end-products; Carb: carbamylation; PC: phosphorylcholine; Met: Methylation; Nitro: nitrated; CWPS: pneumococcal cell wall polysaccharide (contains PC antigen)

In addition, we assessed the lymphoma antibody binding to a wide range of known and exploratory antigens using a custom designed microarray platform covering more than 400 full-length human proteins. None of the tumour-derived lymphoma antibodies showed any strong specific binding to any of the proteins included. In contrast, the lymphoma monoclonal antibody from patient RAL01, RAL09 and the clones from patient RAL10 (RAL10:C84 and RAL10: C214) displayed strikingly high polyreactivity with strong signals to a wide range of unrelated antigens (Fig. 4). Notably these polyreactive mAbs bound 27–70% of the antigens on the array as compared to 86% positivity for the positive controls JB40 and ED38, and 0.2–1.3% for the negative control mAbs as well as for the DLBCL mAbs derived from the patients RAL03 (1.6% binding) and RAL04 (0.9% binding). Comparing the two monoclonal antibodies from patient RAL10, RAL10:C84 displayed higher polyreactivity compared to RAL10:C214 (71% vs. 27% array positivity, respectively). Furthermore, we investigated the binding of the tumour derived antibodies to several autoantigens (Table 2; Figs. 4 and 5), including a panel of antigens modified by post-translation modifications including citrullination, carbamylation, malondialdehyde (MDA) / malondialdehyde acetaldehyde (MAA) modification and methylation. Similarly, the lymphoma antibodies from patient RAL01 and RAL10 showed binding to rabbit IgG but also binding to unmodified BSA (Fig. 4). Notably, all the lymphoma antibodies were negative in the CCP2 IgG ELISA and only the RAL01-derived clone showed any binding to Fc - consistent with RF activity - in comparison to a control surface (Fig. 3). Interestingly, several lymphoma antibodies displayed binding to MDA modified and MAA modified proteins (Fig. 5). Patient RAL01, RAL03, RAL09 and RAL10 lymphoma antibodies showed binding to MDA-LDL, MDA/MAA-BSA and MAA-HSA protein (Fig. 5). However, some of the clones also showed binding to other antigens. The lymphoma antibodies generated from patient RAL03 and RAL09 showed more specific binding to MDA/MAA by ELISA, both towards LDL and/or HSA modified antigen. Due to spontaneous break-down of MDA to acetaldehyde, malondialdehyde modification can generate both linear (MDA) and circular fluorescent (MAA) adducts during the standard modification protocols. Therefore, we also performed an experiment adding cyanoborohydride (SCBH) during the modification, which blocks the formation of MAA adducts (Supplemental Fig. 7). This significantly reduced the binding of RAL03 and RAL09 derived mAbs which indicate preferential MAA binding in line with what has been seen for a majority of previously identified MDA/MAA autoreactive RA-derived clones [33]. Two of these well-studied anti-MDA/MAA clones, G07 (MAA reactive) and F04 (MDA reactive) [15, 33], were used for controls in the assays and the RAL03 clone displayed comparable binding pattern as G07, albeit with higher MDA-LDL binding.

**Table 2:** An overview of reactivities for the lymphoma BCR antibodies

	Control mAbs^*^							RA DLBCL mAbs					
	JB40	ED38	MGO53	E02	B09	G07	F04	RAL01:C22	RAL03:C109	RAL04:C124	RAL09:C9	RAL10:C214	RAL10:C84
HEp2 ANA	*N/A*	*N/A*	**-**	**-**	**++**	*-*	*-*	**-**	**-/+**	**-**	**-/+**	**-**	**-**
Array #	**++++**	**++++**	**-**	**-**	**-**	*N/A*	*N/A*	**++++**	**-**	**-**	**++++**	**++**	**++++**
SMB assay	**++++**	**+++**	**-**	*-*	*-*	*-*	*-*	**+**	**-**	**-**	**+**	**++++**	**++++**
CCP2	*N/A*	*N/A*	*-*	*-*	**++++**	*-*	*-*	**-**	**-**	**-**	**-**	**-**	**-**
Rabbit IgG €	*N/A*	*N/A*	*-*	*-*	*-*	*-*	*-*	**++**	**-**	**-**	**-/+**	**-**	**++**
MDA-LDL	*N/A*	*N/A*	*-*	*-*	*-*	**+++**	**++++**	**++++**	**++++**	**-**	**++++**	**++++**	**++++**
Nitro-LDL	*N/A*	*N/A*	*N/A*	*-*	*N/A*	**-**	**-**	**-**	**-**	**-**	**+**	**++++**	**++++**
CWPS	*N/A*	*N/A*	*N/A*	*-*	*N/A*	**-**	**-**	**-**	**-**	**-**	**-**	**++**	**++**
Carb-BSA	*N/A*	*N/A*	*N/A*	*-*	*N/A*	**-**	**-**	**+**	**-**	**-**	**-**	**+++**	**++**
AGE-BSA	*N/A*	*N/A*	*N/A*	*-*	*N/A*	**-**	**-**	**+**	**-**	**-**	**-**	**+++**	**++**
PC-BSA	*N/A*	*N/A*	*N/A*	*-*	*N/A*	**-**	**-**	**-**	**-**	**-**	**-**	**+++**	**++**
Met-BSA	*N/A*	*N/A*	*N/A*	*-*	*N/A*	**-**	**-**	**+++**	**-**	**+++**	**++**	**+++**	**++**
MDA-BSA	*N/A*	*N/A*	*-*	*-*	*-*	**+++**	**++++**	**+++**	**++++**	**-**	**+**	**+++**	**+++**
MAA-BSA	*N/A*	*N/A*	*-*	*-*	*-*	**+++**	**++++**	**++**	**+++**	**-**	**+++**	**+++**	**+++**
MAA-HSA	*N/A*	*N/A*	*N/A*	*-*	*N/A*	**++++**	**++++**	**++**	**++++**	**-**	**++++**	**++++**	**++++**

# Polyreactivity by a broad solid phase antigen array

€ Rabbit IgG ELISA was used to assess potential rheumatoid factor (RF) activity

*SMB* Soluble membrane protein polyreactivity assay, *ANA* Anti-nuclear autoantibodies, *CCP2* Cyclic citrullinated peptide 2, *MDA* Malondialdehyde,* MAA* Malondialdehyde acetaldehyde, *AGE* Advanced glycan end-products, *Carb* Carbamylation, *PC* Phosphorylocholine, *Met* Methylation, *LDL* Low density lipoprotein, *Nitro* Nitrated, *CWPS* pneumococcal cell wall polysaccharide

* Control mAbs have previously been tested and screened. JB40 and ED38 have previously been confirmed to show polyreactive binding [30], MGO53 [30] and E02 (1362:01E02) have been used as a negative control for citrulline and MDA binding in multiple studies The CCP2 + ACPA B09 (1325:01B09) bind citrullinated and acetylated peptides and proteins and is ANA+ through acetylated-histone binding [32, 38]. G07 (146+:01G07) and F04 (1276:01F04) have demonstrated specific binding to MDA and MAA modified proteins in previous studies [15, 33]

Patient RAL04 lymphoma antibody showed binding to methylated antigen and low binding to the other evaluated antigens. No direct correlation between EBV positivity and polyreactivity could be observed, as only RAL10 out of the highly polyreactive clones were detected as EBV positive. Interestingly, the second monoclonal antibody from patient RAL10 (RAL10:C214), also showed broad antigen binding but with lower fluorescent intensity compared to RAL10:C84. None of the patient tumour derived antibodies showed any significant anti-nuclear antibody (ANA) reactivity by assessment with a standardized Hep-2 cell immunofluorescence assay (Supplemental Fig. 8). Hence, in conclusion two clones showed high specificity to either MDA/MAA modified antigens (RAL03) or methylated protein (RAL04), while the other clones displayed different levels of polyreactive or promiscuous autoreactivity (reactivity summary is provided in Table 2) .

## Discussion

Here we demonstrate that tumour BCRs from DLBCL in RA patients display a high degree of autoreactivity with polyreactivity or reactivity directed towards oxidation-associated post-translational modifications mediated by malondialdehyde. Half of the tumour B cells expressed B cell receptors carrying high level of somatic hypermutations and 30% had low levels. Yet, no association was seen between the level of somatic hypermutation and the level of polyreactivity. Importantly, we demonstrate evidence for both antigen-selection of the BCR clonotype and intra-clonotype evolution suggesting a continuous antigen-dependent promotion of the tumours in RA-DLBCL patients. We did not see any shared gene-usage preference. While other studies have shown an enrichment for VH4-34 in DLBCL, none of the RA-DBCL tumour BCRs were encoded by VH4-34 and instead a majority were VH3 family encoded [39]. Among the 22 RA-DLBCL patients included for BCR sequencing, we detected EBV in the tumour tissue for three patients (14%) which is slightly higher compared to previous reports of DLBCL in general (5–9%) [40] but in line with a study of DLBCL in elderly [41]. EBV has been implicated as a critical risk factor for development of several autoimmune diseases [42]. Although the most polyreactive DLBCL BCR in our study was from an EBV+ tissue, our material was too limited to make any conclusions about EBV association with lymphoma BCR antigen-reactivity. Yet, we acknowledge that no clear enrichment for EBV positivity was indicated for RA-DLBCL based on our study.

Here we investigated the RA DLBCL lymphoma BCR antigen reactivity by re-expressing lymphoma BCRs as recombinant monoclonal antibodies. Three of the lymphoma antibodies showed binding in our in-house polyreactivity assay and two antibodies from patient RAL01 and RAL10 showed strikingly high polyreactivity in the antigen array. Interestingly, the lymphoma antibody RAL10:C84 with leucine (L) 37 showed more polyreactive binding in multiple assays compared to RAL10:C214 with valine (V) 37 (germline version), despite originating from the same tumour and only differing by one amino acid with quite similar biochemical properties. Notably, this residue displayed variability within the dominating lymphoma clonotype with both leucine and valine occurring in multiple expanded sequences, which may indicate an evolutionary drive for the lymphoma BCR in relation to their polyreactivity. Antibody polyreactivity can be defined as low affinity antibody binding to multiple unrelated antigens. The underlying biochemical properties mediated have been suggested to include surface flexibility and/or high charge or hydrophobicity in the binding site [43]. It is well established that both the immature B cell and the mature IgM+ repertoire contains a large proportion of polyreactive clones [30, 44].

B cells with polyreactivity may be involved in the first-line of defense in the immune system by providing early protection against pathogens and enhancing presentation to T cells [43], however they can also show overlap with anti-nuclear autoreactivity and contribute to autoimmunity as precursor for more specific autoreactivity [45]. B cells with polyreactivity have been estimated to constitute up to 20% of the IgG+ memory population [46] and different levels of polyreactivity can also be observed in specific viral response such as among anti- human immunodeficiency virus (HIV) antibodies [47]. RA patients have been demonstrated to have an increased proportion of polyreactive B cells in the naïve repertoire, which has been proposed to be due to defects in B cell tolerance [48]. Furthermore, we have previously reported high proportion of polyreactive class-switched clones in RA lungs [36] and gingiva tissue [37], two mucosal sites that have been suggested to be important for the break of tolerance in this disease. In the current re-analysis of clones, we detect 20% and 21% polyreactive clones in gingiva and lung B cells which could be compared with the 9% in bone marrow plasma cells, which is in line with previous literature [46].

Monoclonal polyreactivity is often assessed by ELISA, antigen arrays or flow cytometry to control antigens. In addition, some polyreactive clones, but not all, bind non-specifically to synthetic hydrophobic surfaces, and for example show binding to empty ELISA wells. While a traditional method is using a combination of ELISAs for binding to dsDNA, insulin and LPS, we here primarily used a soluble membrane protein (SMP) assay that we have previously developed [32]. Notably SMP-polyreactivity correlated for most clones with promiscuous binding patterns in other assays. Intriguingly, the tumour derived antibody from patient RAL01 showed high polyreactive binding in the antigen array, but displayed lower promiscuity in the in-house polyreactivity assay. These discrepancies observed for the RAL01 patient derived clone may suggest that different types of unspecific interactions (e.g. hydrophobic vs. charge based) are captured with different assays. Moreover, none of the polyreactive clones showed any significant ANA reactivity. Since some protein modifications change the hydrophobicity and surface charge of proteins and induce structural changes that expose aliphatic residues, there might be some overlap between polyreactivity and apparent autoreactivity towards certain protein modifications. Yet, B cell clones can also maintain polyreactive properties while simultaneously gaining affinity for specific epitopes, as has been demonstrated for antibodies in HIV responses, where a large proportion of highly hypermutated neutralizing anti-HIV antibodies also have polyreactive binding to lipids and protein self-antigens [47].

In the current study, we found high prevalence of positivity towards malondialdehyde modified protein, in particular MDA-LDL. All three polyreactive lymphoma antibodies showed binding to MDA/MAA modified antigens. Two of these RAL01 and RAL09, displayed high reactivity for MDA-LDL but some polyreactivity with unspecific binding in other assays. Notably, the two lymphoma antibodies derived from patients RAL03 and RAL04 which were not polyreactive in the in-house assay or antigen array, displayed a more specific binding to MDA/MAA modified antigen or methylated antigen, respectively.

While IgM antibodies binding to MDA/MAA protein adducts, in particular in the context of oxidized low density lipoprotein, are part of the natural antibody repertoire, prevalent at birth [49–51], and with potential homeostatic properties [52], IgG anti-MDA/MAA are associated with inflammation. We and others have found that IgG anti-MDA/MAA antibodies are increased in RA patients and that the antibody levels correlate with disease activity and inflammatory markers [15, 17].While IgG anti-MDA is not disease-specific and is not routinely measured clinically it is investigated as an interesting potential biomarker for RA. Reactive MDA that results in MDA protein modification is generated during oxidative stress and inflammation can be hypothesized to increase the MDA-antigen burden and drive the elevation of anti-MDA antibodies [53]. In vitro and in vivo studies using patient-derived monoclonal antibodies have shown that IgG anti-MDA can activate inflammatory pathways which may further increase generation of the MDA/MAA adducts, thereby potentially creating a vicious circle [15, 53–55]. Our previous investigations show that in RA, most anti-MDA antibodies predominantly bind the MAA adducts generated in the presence of acetaldehyde. Anti-MDA/MAA reactive B cells have also been found in different B cell compartments and tissues, and have been shown to display different level of SHM but binding is retained in germline-converted versions [33]. Interestingly, a subset of chronic lymphocytic leukemia (CLL) has also been shown to carry BCRs with anti-MAA reactivity [56, 57]. None of the lymphoma antibodies were positive for the RA hallmark anti-CCP2 autoreactivity or displayed any specific RF properties. This suggests that the lymphoma origin is distinct from classical RA-specific autoimmunity. However, our demonstration of anti-MAA reactivity in RA-DLBCL patients may instead provide a potential link between inflammation and oxidative stress and the increased risk of lymphoma development in RA patients mediated by a pathway promoting ongoing MDA-antigen exposure and BCR activation.

The majority of patient derived lymphoma BCRs had high or moderate levels of SHM and thereby resembled germinal center experienced B cells. Hence, our results were consistent with previous studies showing that DLBCL BCRs can be both unmutated and mutated [39, 58, 59]. Interestingly, both clonotypes with more specific MDA reactivity carried SHMs. For many of the lymphomas RAL01, RAL03 and RAL07 with SHM, we could detect an accumulation of amino acid replacements in the CDR regions compared FW regions, indicating antigen-selection.

For three tumour clonotypes we observed intra-clonal amino acid variation, indicating ongoing clonal evolution. Notably, in general we detected significant sequence variation within the tumour clonotype, but it is important to acknowledge that the multiplex PCR and Illumina methodology is associated with PCR and sequencing errors that may result in apparent sequence variability. When using RNA based sequencing this can be more easily controlled for by molecular barcodes, but due to the high RNA fragmentations, we here had to rely on DNA as starting material. However, in the current study, even though not all biopsies might be pure tumour tissue, we amplify a mostly monoclonal BCR tissue which results in a very large number of reads within the dominating clonotype. Careful analysis of the nucleotide variations within the tumour BCRs, demonstrated a number of unique sequences with single nucleotide variations present at low representation, which we conclude reflects PCR and sequencing errors. In contrast, some sequences were more expanded in terms of read counts, although still at lower proportion that the most prevalent sequence which are likely reflecting intra-clonal variations. Yet, differences in DNA degradation and PCR amplification bias can influence the exact proportions of sequence variances and true intraconal variation can only be determined by single cell methods.

Amino acid analysis confirmed recurrent variations in a large proportion of the sequences for some of the tumour tissues. Importantly, intra-clonal variation and ongoing BCR SHM in the tumours have previously been shown to be associated with poorer prognosis [59].

Furthermore, it is important to address that the size of the cohort included for BCR sequencing is relatively small with nine patients. However, the RA-DLBCL patient cohort is unique and thereby provided us with a unique opportunity to investigate the BCR sequences and their binding in the context of autoantigens.

In conclusion, our study revealed that lymphoma BCR from RA-DLBCL patients can display high polyreactivity, and that there may be an evolutionary selection within the tumour suggested by the intraclonal diversity observed. We also show that for some patients, the tumour B cell receptor was not promiscuous in its binding and instead showed a more specific binding to MDA/MAA modified protein. We hypothesize that high and chronic inflammation in RA patients, which has been linked to an increased risk of lymphoma development, may contribute to the formation of modified self-antigens such as MDA/MAA adducts. This may drive chronic excessive activation of autoreactive anti-MDA+ and polyreactive B cells. With the inflammation and RA-associated reduced tolerance, these activated B cells can enter germinal centers and with persistent stimulation over time can be hypothesized to be at higher risk of acquiring features associated with malignant transformation into DLBCL.

## Supplementary Information

Supplementary Material 1.

## Data Availability

Data can be made available upon reasonable request.
