# Supplementary material for: Diffuse large B cell lymphoma in rheumatoid arthritis express polyreactive and autoreactive BCRs binding protein modifications
Source: Arthritis Res Ther. 2026 Oct 27;28:188. doi: 10.1186/s13075-026-03937-0 (PMC13711851; doi:10.1186/s13075-026-03937-0)
Supplement: Supplementary file 1 [file 13075_2026_3937_MOESM1_ESM.pdf]

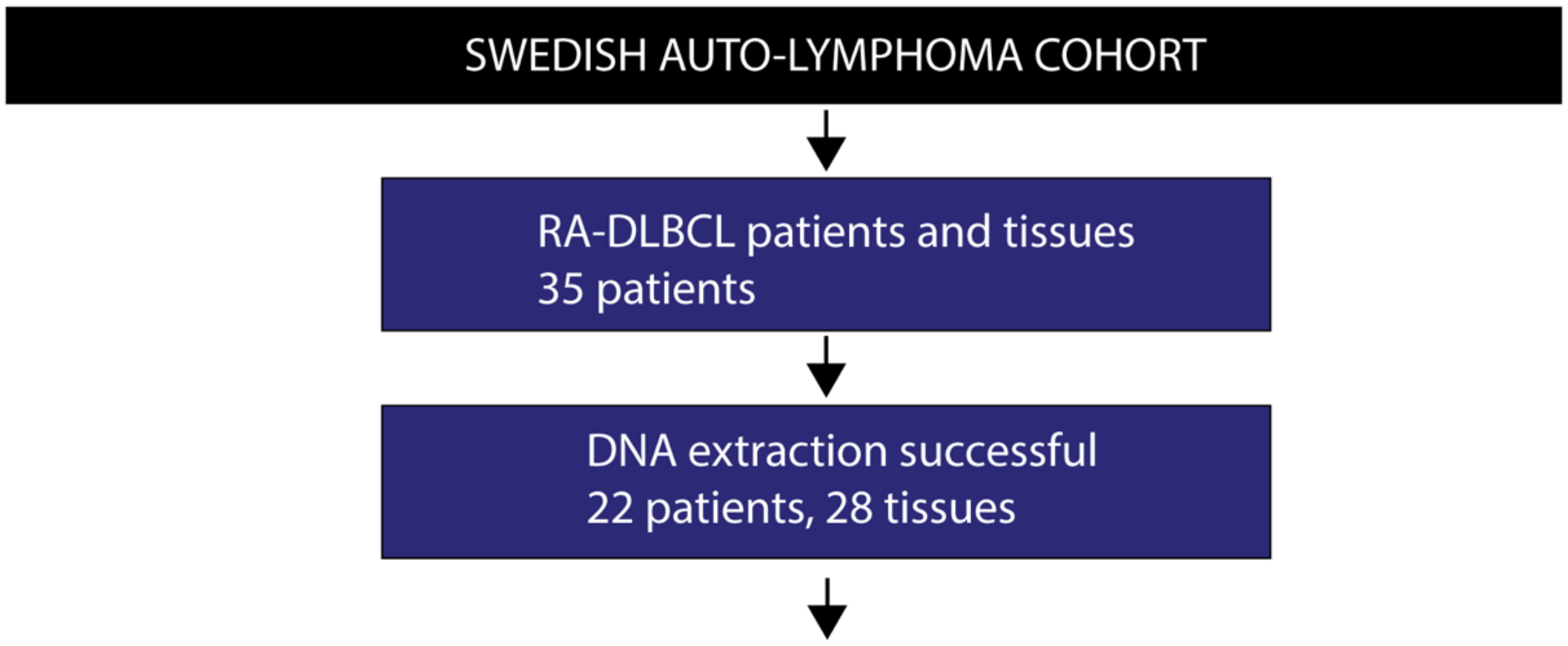

DNA EXTRACTION SUCCESSFUL	biopsies	VH PCR & SEQUENCING SUCCESSFUL	biopsies	VL PCR & SEQUENCING SUCCESSFUL	biopsies	mAbs EXPRESSION SUCCESSFUL		mAb IDs	
RAL01		RAL01		RAL01		RAL01		**RAL01:C22**	
RAL02									
RAL03		RAL03		RAL03		RAL03		**RAL03:C109**	
RAL04	A*, B	RAL04	B	RAL04		RAL04		**RAL04:C124**	
RAL05		RAL05							
RAL06		RAL06#		RAL06					
RAL07	A, B	RAL07	A	RAL07	A	(RAL07)€			
RAL08		RAL08							
RAL09		RAL09		RAL09		RAL09		**RAL09:C9**	
RAL10	A*, B*, C*	RAL10	A, B, C	RAL10	A, B	RAL10	2 clones	**RAL10:C84**	**RAL10:C214**
RAL11	*								
RAL12									
RAL13									
RAL14									
RAL15									
RAL16	A, B								
RAL17									
RAL18	A, B								
RAL19									
RAL20									
RAL21									
RAL22									

# no dominating VH identified

€ poor expression

** EBV positive by QPCR*

Supplemental Figure 1. Overview of the RA-DLBCL patients and BCR sequencing results

The figure visalizes the workflow and results of the DNA extraction and BCR sequencing of formalin-fixed paraffin-embedded tumour tissues from RA patients with DLBCL.

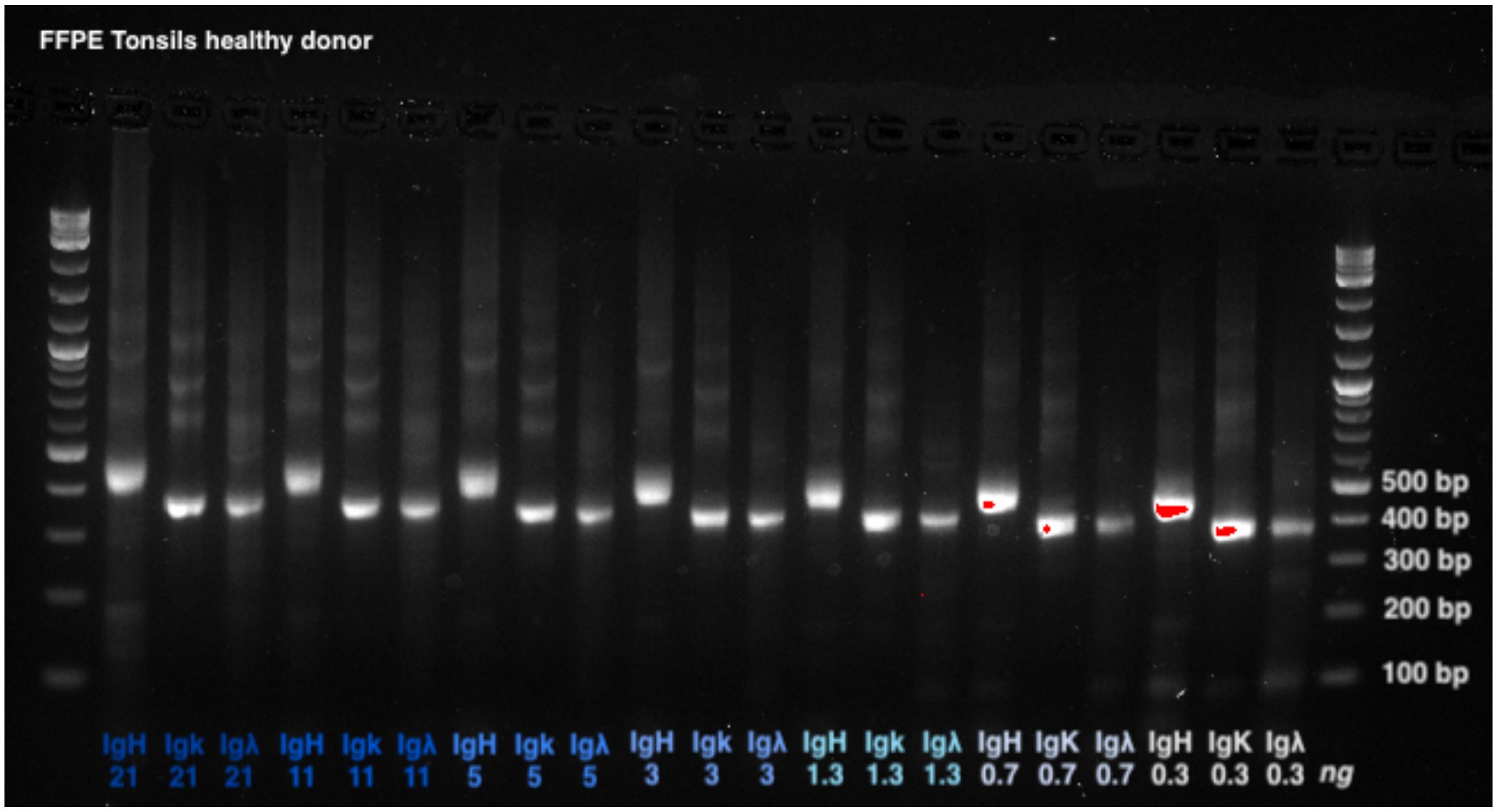

Supplemental Figure 2. Dilution of FFPE DNA with nuclease free water improved PCR amplification as examplified by healthy tonsil FFPE DNA.

Here we demonstrate that dilution of FFPE tonsil DNA improved PCR amplification for heavy chain (IgH), kappa (Igk) and lambda (Ig λ) light chain with comparison starting at 21 ng FFPE DNA input, down to 0.3 ng FFPE DNA input.

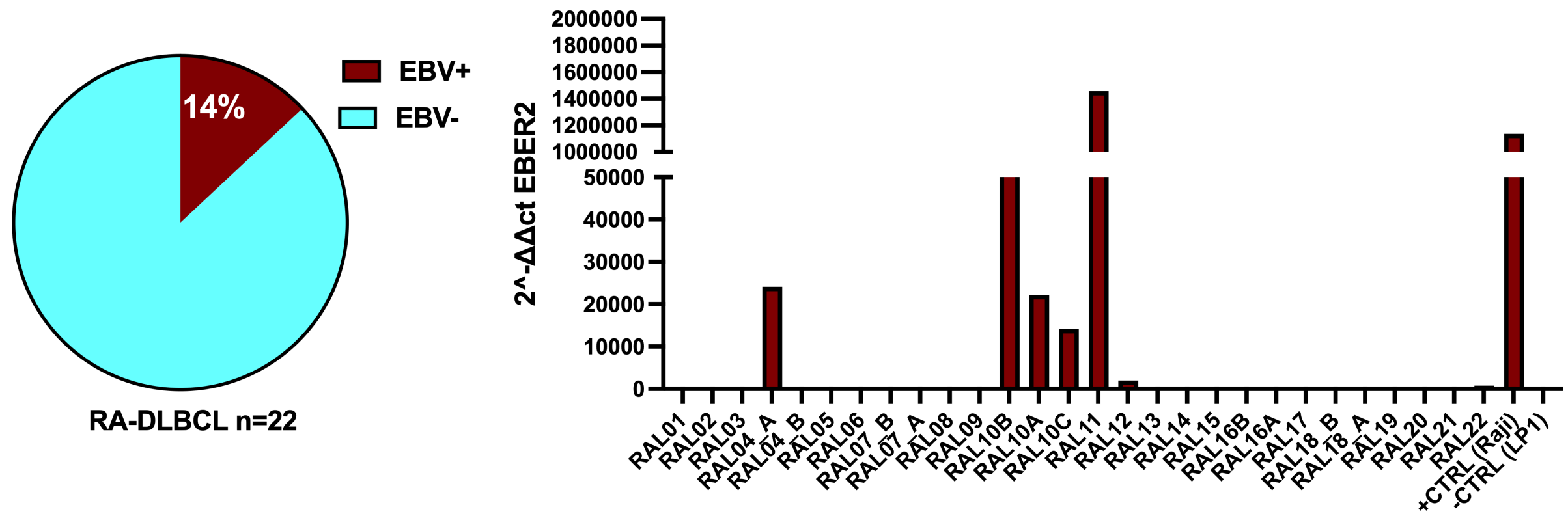

Supplemental Figure 3. EBV detection in tumour tissue from RA-DLBCL patients.

Epstein Barr virus (EBV) infection status of the tumour tissue from RA-DLBCL patients (n = 9) was determined by an in-house qPCR protocol (quantification of EBER2). A positive control (+CTRL; Raji cell line) and negative control (-CTRL; LP1) were included as reference.

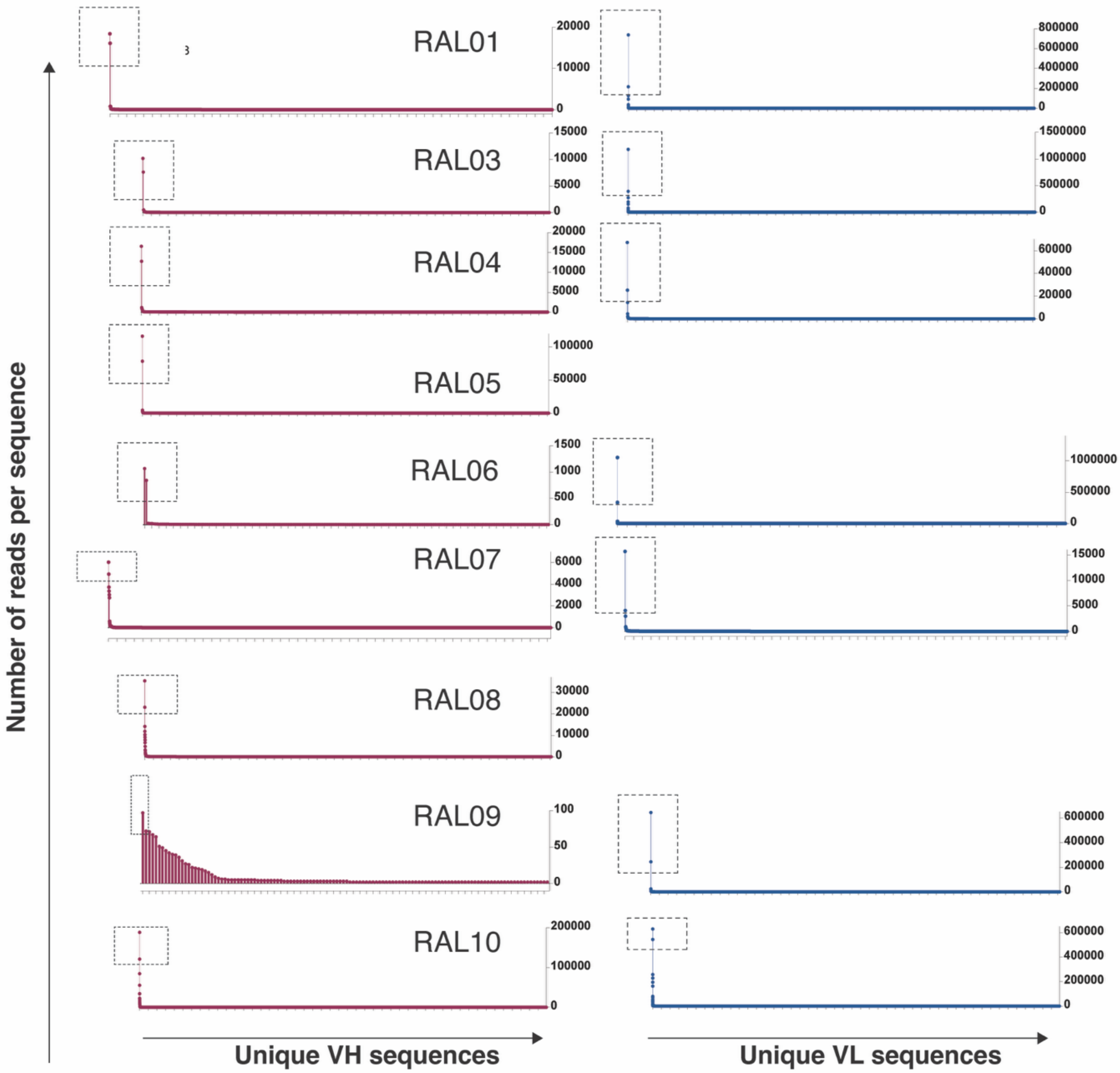

Supplemental Figure 4. Number of unique sequences and reads within the dominating clonotype lineage in DLBCL BCR sequencing

The figure is visualizing all unique nucleotide sequences within the dominating clonotype by VH-JH/VL-JL gene usage and CDR3 length from Illumina sequencing of biopsies from 10 RA-DLBCL patients. The x-axis is showing unique sequences, and the y-axis is showing the number of sequence reads (counts of duplicated sequences, dupcounts). Highest read counts are highlighted in the boxes. The two VH/VL sequences with highest read counts are identical despite a few nucleotide in the start or end due to primer identification and masking. The numbers are presented in Supplemental Table 7 and 8.

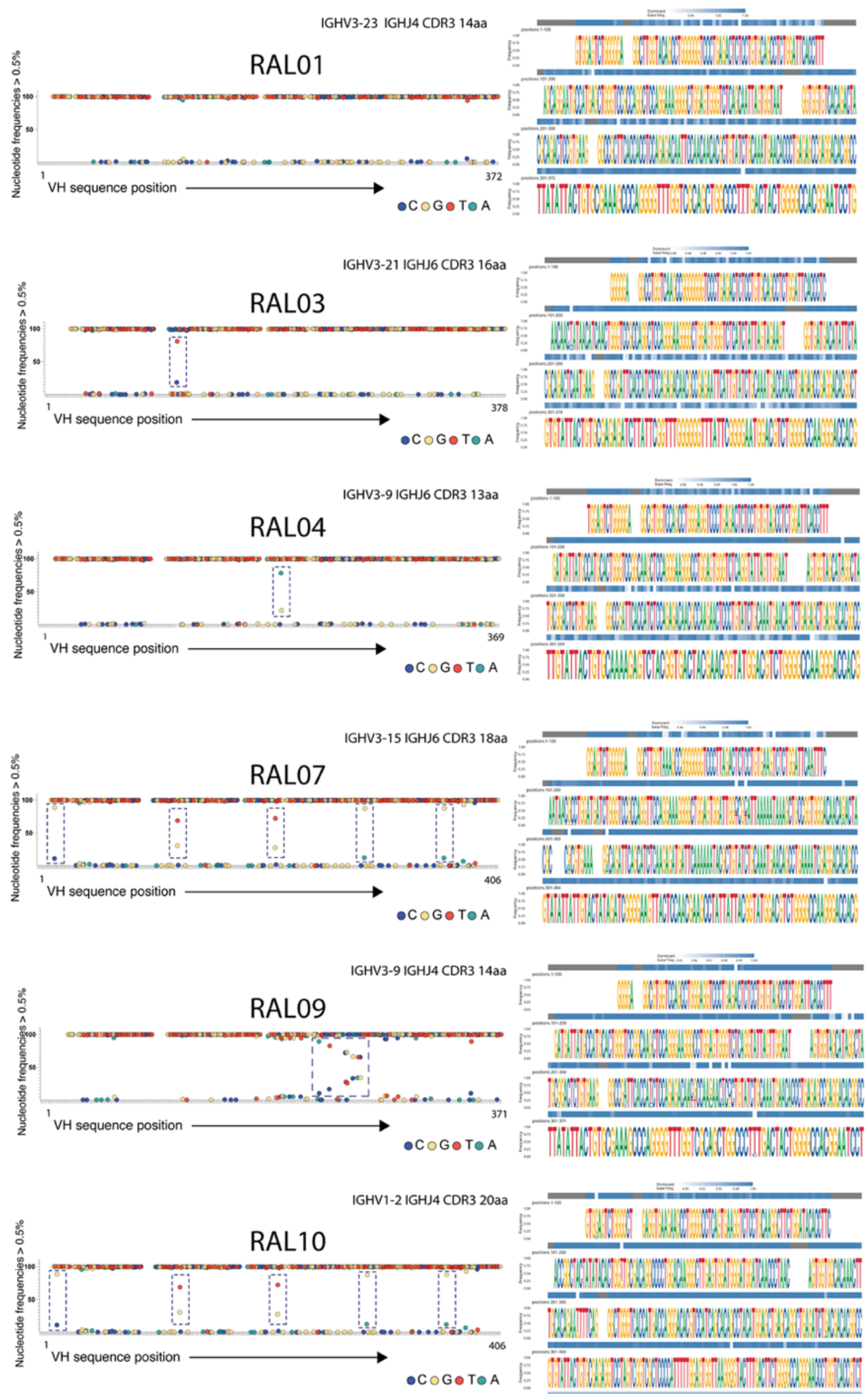

Supplemental Figure 5. Nucleotide variation within the dominating clonotype

The figure is visualizing the nucleotide variation position by position within the dominating VH clonotype based on V-J gene usage and CDR3 length from BCR tumour tissues of six RA-DLBCL patients (RAL01, RAL03, RAL04, RAL07, RAL09 and RAL10) from which monoclonal antibodies were generated based on the most dominating read. For RAL10 the two most frequent VH sequences within the clonotype were used to generate two monoclonals. The left panel is showing the proportion of C/G/A/T in each position within all VH reads from the clonotype (values above >0.5%). The right panel shows the sequence alignment with stacked letter logo-visualization of the nucleotides. Low variance should be considered to come from PCR or sequencing error, while high proportion of variance are likely inter-clonal variation and BCR evolution.

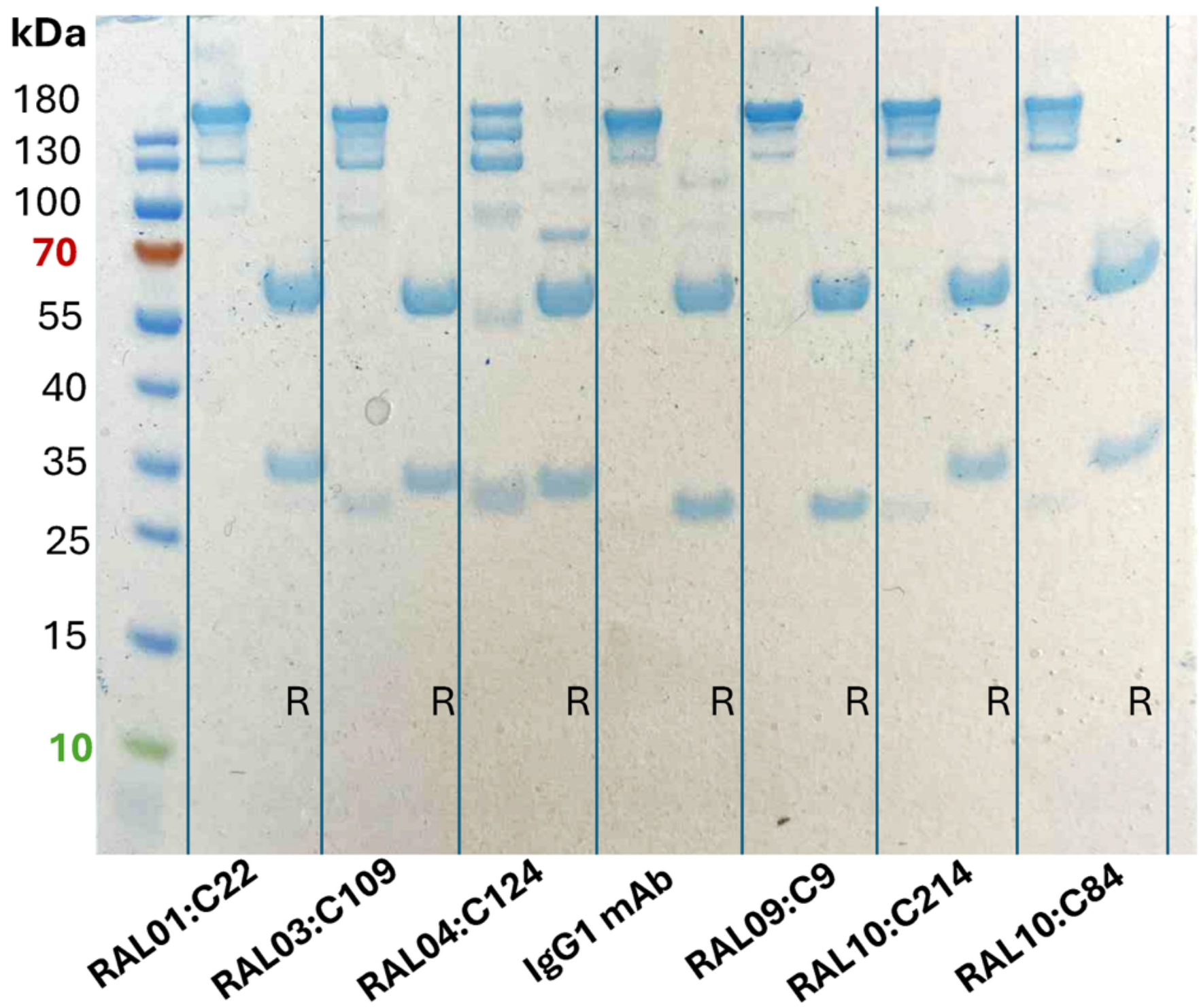

Supplemental Figure 6. SDS PAGE of recombinant tumour monoclonal antibodies.

To verify that the recombinant tumour monoclonal antibodies produced are of good quality a SDS-PAGE was done using 3 μg of each monoclonal antibody with and without reducing (R) conditions.

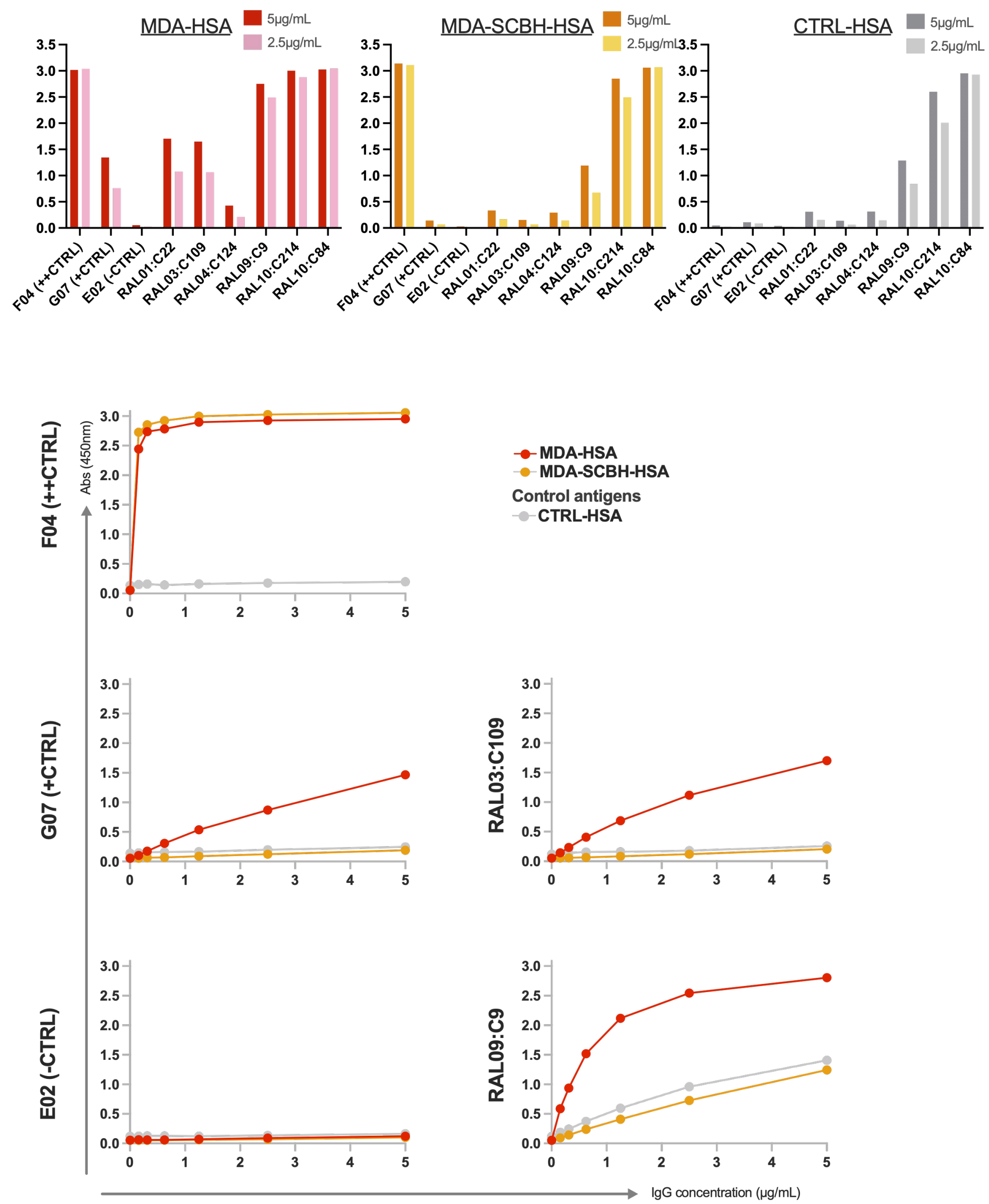

Supplemental Figure 7. Evaluation of binding to MAA adducts compared to MDA adducts

Human serum albumin (HSA) was malondialdehyde (MDA) modified with or without the presence of chemical reducing agent sodium cyanoborohydride (SCBH) that blocks the formation of fluorescent MAA adducts (dihydropyridine-lysine) as previously described [1]. Binding of 6 RA-DLBCL tumour BCR derived recombinant hIgG1 monoclonal antibodies to MDA-HSA (with both MDA and MAA adducts), MDA-SCBH-HSA (with only MDA adducts) and control unmodified HSA, was assessed by ELISA at indicated IgG dilutions. Binding was compared to that of two previously reported positive control mAbs, 1276:01F04 (F04, MDA-reactive) and 146+:01G07 (G07, MAA-reactive) [2] as well as the negative control clone 1362:01E02 (E02, no known binding) [3]. Both the RAL03 and RAL09 patient derived clones show preferential MAA binding over MDA.

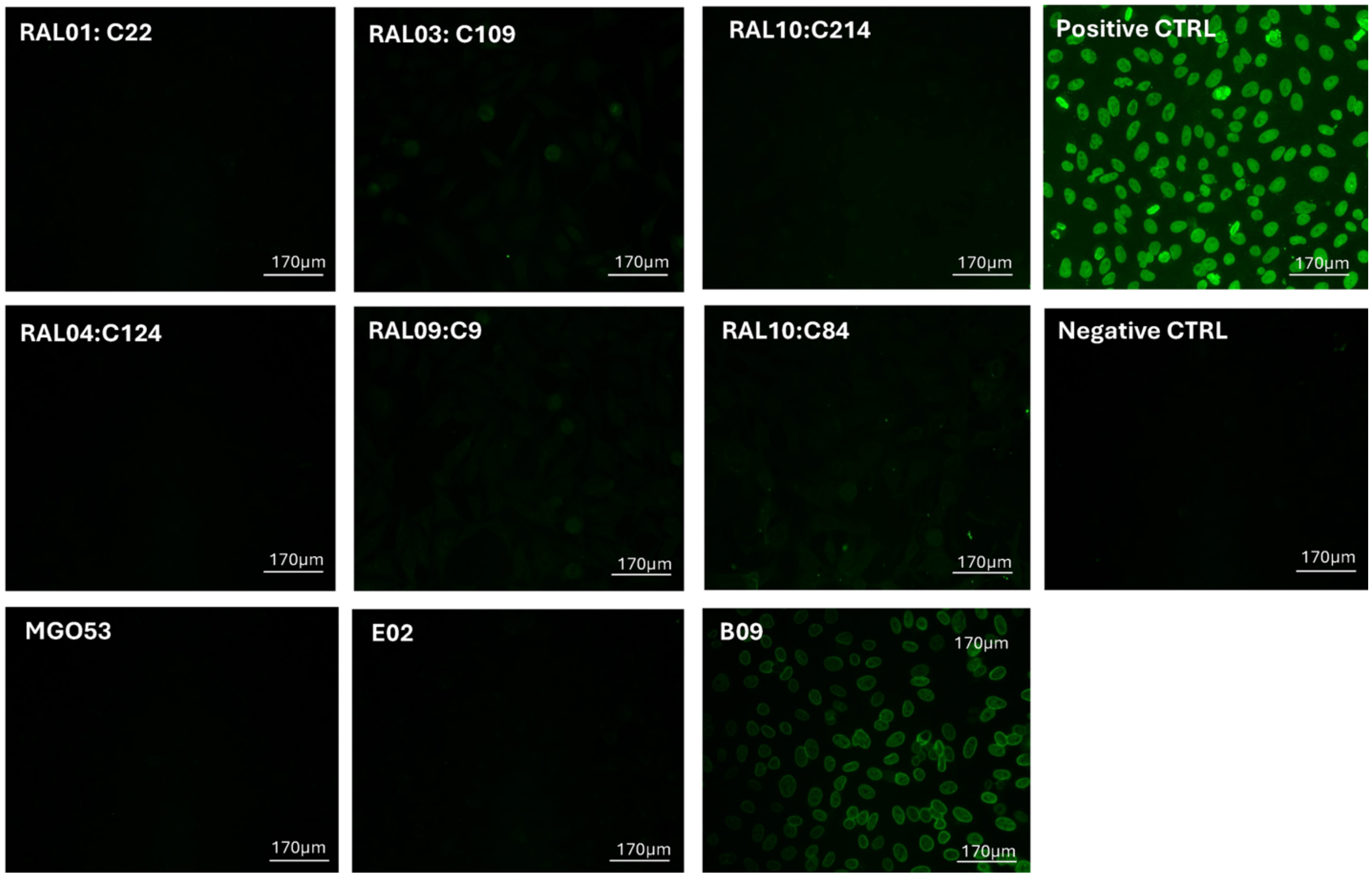

Supplemental Figure 8. Tumour BCR dereived mAbs do not display anti-nuclear antibody (ANA) reactivity by immunofluorescence

Antinuclear antibody (ANA) binding was determined by immunofluorescence staining to NOVA lite Hep-2 ANA slides and visualized by ECHO Revolve fluorescent microscope. The mAbs for the ANA staining were tested at 10 µg/ml. The commercial assay included an internal positive and negative control. We also included recombinant monoclonal IgG1 expressed in the same system as controls: 1325:01B09 as positive control (B09, anti-CCP2 positive ACPA mAb with additional reactivity to acetyl-lysine/acetylated histones and previously reported ANA positivity) [4, 5] and 1362:01E02 (E02) [3] and MGO53 [6], as a negative controls (both clones have no known reactivity to any post-translationally modified or native antigens).

Supplemental Table 1. An overview of RA-DLBCL patients with tumour BCR sequencing

	**Seropositive#**	**DLBCL subtype€**	**Extranodal engagement**	**Ann Arbor**	**EBV***	**Lymphoma relapse first 5 years**
**RAL01**	Yes	non-GCB	Yes	IV	Neg	No
**RAL03**	Yes	GCB	Yes	IV	Neg	No
**RAL04**	Yes	non-GCB	Yes	IV	Neg**	No
**RAL05**	No	non-GCB	No	I	Neg	No
**RAL06**	No	N/A	Yes	IE	Neg	No
**RAL07**	Yes	N/A	Yes	IV	Neg	No
**RAL08**	Yes	N/A	No	III	Neg	Yes
**RAL09**	Yes	GCB	Yes	IV	Neg	No
**RAL10**	Yes	N/A	Yes	II	Pos	No

anti-CCP2 IgG and/or RF IgM serum positivity.
€ DLBCL subtype according to Hans algorithm. (8)
* Epstein Barr virus in lymphoma tissue analyzed by qPCR of EBER2.
**Another tumour tissue sample from patient RAL04 was positive for EBV by qPCR. The tissue which was sufficient for BCR library preparation was not positive for EBV by qPCR.

Supplemental Table 2. Clinical characteristics in RA-DLBCL patients with tumour sequencing

	N=9
Female	4 (44%)
Age at RA onset, median, years (range)	59 (16-70)
Age at lymphoma diagnosis/inclusion, median, years (range)	72 (61-79)
RA duration at lymphoma diagnosis, median, years (range)	21 (2-45)
RF and/or CCP-positivity	7 (78%)
RA treatment, ever before lymphoma[#]	
Any DMARD, number of patients (%)[€]	8 (89%)
Methotrexate	7
Hydroxychloroquine	4
Leflunomide	3
Cyklosporine A	1
Sulfasalazine	1
Adalimumab	2
Etanercept	1
Abatacept	1
Rituximab for RA	0
Prednisolone	3

DMARD: Disease modifying anti-rheumatic drug
[#]At lymphoma diagnosis DMARD treatment was temporarily or permanently stopped in all patients
[€]Number of DMARDs ever used per patient differed between 1 and 6

Supplemental Table 3. PCR primers for preparing BCR libraries.

Oligo pool	Individual oligo	Sequence 5'-3'	Comment
i5-VH pool	i5-HV1_1_70_Fwrd	ACACTCTTTCCCTACACGACGCTCTTCCGATCTSCAGCTGGTGCAGTCTGG	IGHV1 forward primer, with i5 adapter attached
	i5-HV1/3/5_70_Fwrd	ACACTCTTTCCCTACACGACGCTCTTCCGATCTGTGCAGCTGGTGGAGTCTG	IGHV1/3/5 forward primer, with i5 adapter attached
	i5-HV2_Fwrd	ACACTCTTTCCCTACACGACGCTCTTCCGATCTTCACCTTGAAGGAGTCTGG	IGHV2 forward primer, with i5 adapter attached
	i5-HV4_1_Fwrd	ACACTCTTTCCCTACACGACGCTCTTCCGATCTTGCAGCTGCAGGAGTCG	IGHV4 forward primer, with i5 adapter attached
	i5-HV4_2_Fwrd	ACACTCTTTCCCTACACGACGCTCTTCCGATCTGTGCAGCTACAGCAGTGG	IGHV4 forward primer, with i5 adapter attached
	i5-HV6_Fwrd	ACACTCTTTCCCTACACGACGCTCTTCCGATCTGTACAGCTGCAGCAGTCA	IGHV6 forward primer, with i5 adapter attached
i7_JH	i7_JH_Rev	AGACGTGTGCTCTTCCGATCTCTTACCTGAGGAGACGGTGACC	Universal JH reverse primer
VK pool	HSCK1-F	GGGCCCAGGCGGCCGAGCTCCAGATGACCCAGTCTCC	IGKV/1 forward primer, with 5' extension adapter
	HSCK24-F	GGGCCCAGGCGGCCGAGCTCGTGATGACYCAGTCTCC	IGKV2/4 forward primer, with 5' extension adapter
	HSCK3-F	GGGCCCAGGCGGCCGAGCTCGTGWTGACRCAGTCTCC	IGKV3 forward primer, with 5' extension adapter
	HSCK5-F	GGGCCCAGGCGGCCGAGCTCACACTCACGCAGTCTCC	IGKV5 forward primer, with 5' extension adapter
VL pool	HSCLam1a	GGGCCCAGGCGGCCGAGCTCGTGBTGACGCAGCCGCCCTC	IGLV forward primer, with 5' extension adapter
	HSCLam1b	GGGCCCAGGCGGCCGAGCTCGTGCTGACTCAGCCACCCTC	IGLV forward primer, with 5' extension adapter
	HSCLam2	GGGCCCAGGCGGCCGAGCTCGCCCTGACTCAGCCTCCCTCCGT	IGLV forward primer, with 5' extension adapter
	HSCLam3	GGGCCCAGGCGGCCGAGCTCGAGCTGACTCAGCCACCCTCAGTGTC	IGLV forward primer, with 5' extension adapter
	HSCLam4	GGGCCCAGGCGGCCGAGCTCGTGCTGACTCAATCGCCCTC	IGLV forward primer, with 5' extension adapter
	HSCLam6	GGGCCCAGGCGGCCGAGCTCATGCTGACTCAGCCCCACTC	IGLV forward primer, with 5' extension adapter
	HSCLam78	GGGCCCAGGCGGCCGAGCTCGTGGTGACYCAGGAGCCMTC	IGLV forward primer, with 5' extension adapter
	HSCLam9	GGGCCCAGGCGGCCGAGCTCGTGCTGACTCAGCCACCTTC	IGLV forward primer, with 5' extension adapter
	HSCLam10	GGGCCCAGGCGGCCGAGCTCGGGCAGACTCAGCAGCTCTC	IGLV forward primer, with 5' extension
CL pool	CJLam1236-NL3	AGAGGAGGAAGATCTAGAGGAACCACCGCCTAGGACGGTCASCTTGGTSCC	Universal IGLJ reverse primer, with 3' extension adapter
	CJLam4-NL3	AGAGGAGGAAGATCTAGAGGAACCACCGCCTAAAATGATCAGCTGGGTTCC	Universal IGLJ reverse primer, with 3' extension adapter
	CJLam57-NL3	AGAGGAGGAAGATCTAGAGGAACCACCGCCGAGGACGGTCAGCTSGGTSCC	Universal IGLJ reverse primer, with 3' extension adapter
CK pool	CJK14-NL3-rev	AGAGGAGGAAGATCTAGAGGAACCACCTTTGATYTCCACCTTGGTCC	Universal IGKJ reverse primer, with 3' extension adapter
	CJK2-NL3-rev	AGAGGAGGAAGATCTAGAGGAACCACCTTTGATCTCCAGCTTGGTCCC	Universal IGKJ reverse primer, with 3' extension adapter
	CJK3-NL3-rev	AGAGGAGGAAGATCTAGAGGAACCACCTTTGATATCCACTTTGGTCCC	Universal IGKJ reverse primer, with 3' extension adapter
	CJK5-NL3-rev	AGAGGAGGAAGATCTAGAGGAACCACCTTTAATCTCCAGTCGTGTCCC	Universal IGKJ reverse primer, with 3' extension adapter
i5-EXT	I5_EXT-RT	ACACTCTTTCCCTACACGACGCTCTTCCGATCTNNNNNGGGCCCAGGCGGCCGAGCTC	i5 adapter attachment primer
i7-LIN	I7_VL_linker	ACTGGAGTTCAGACGTGTGCTCTTCCGATCTAGAGGAGGAAGATCTAGAGGAACCAC	i7 linker forward primer

Primer design ha previously been reported in [7]

Supplemental Table 4. FFPE DNA input

Patient tumour sample	DNA input	Concentration (ng/ul)	DIN
RAL01	5.1 ng	5.1	4.1
RAL03	4.4 ng	4.4	1.7
RAL04	1.6 ng	1.6	Below detection
RAL05	8.8 ng	352.0	2.7
RAL06	67.8 ng	67.8	4.8
RAL07	11 ng	11.2	4.6
RAL08	1.0 ng	103.0	3.6
RAL09	1.0 ng	44.6	5.8
RAL10_A	10.3 ng	720.0	3.6
RAL10_B	49.1 ng	49.1	4.1
RAL10_C	2.7 ng	27.4	2.9
HD tonsil	1.0 ng	180.2	6.5

Supplemental Table 5. PCR primers for adapter attachment

Index	MID ID	Primer sequence
I5_index_S513	TCGACTAG	AATGATACGGCGACCACCGAGATCTACACTCGACTAG ACACTCTTTCCCTACACGACG
I5_index_S515	TTCTAGCT	AATGATACGGCGACCACCGAGATCTACACTTCTAGCTACACTCTTTCCCTACACGACG
I5_index_S516	CCTAGAGT	AATGATACGGCGACCACCGAGATCTACACCCTAGAGTACACTCTTTCCCTACACGACG
I5_index_S517	GCGTAAGA	AATGATACGGCGACCACCGAGATCTACACGCGTAAGAACACTCTTTCCCTACACGACG
I5_index_S518	CTATTAAG	AATGATACGGCGACCACCGAGATCTACACCTATTAAGACACTCTTTCCCTACACGACG
I7_index_N716	TAGCGAGT	CAAGCAGAAGACGGCATACGAGATTAGCGAGTGTGACTGGAGTTCAGACGTGTGCTCTTCCGATCT
I7_index_N718	GTAGCTCC	CAAGCAGAAGACGGCATACGAGATGTAGCTCCGTGACTGGAGTTCAGACGTGTGCTCTTCCGATCT

Supplemental Table 6. Filtering of reads based on general quality metrics

Patient	Before filtering<br>Total reads	Duplication rate	size peak (unfiltered)	After filtering<br>Q20	Q30	Reads passed filters
RAL01	6,383,380	48.4%	330 bp	98.33%	93.28%	5 640 158 (88.36%)
RAL03	17,529,708	45.82%	327 bp	98.13%	92.75%	16 688466 (95.20%)
RAL04	4,715,854	21.39%	47/315bp€	97.75%	86.62%	3 369 590 (71.45%)
RAL05	3,615,416	35.84%	327 bp	96.94%	90.06%	3 289 486 (98.00%)
RAL06	71,123,608	62.20%	317 bp	98.52%	94.17%	68 697946 (96.6%)
RAL07	2,892,194	32.91%	327 bp	98.11%	92.60%	2 692 370 (93.10%)
RAL08	4,903,238	40.58%	317 bp	98.21%	93.03%	4 711 718 (96.09%)
RAL09	19,687,266	47.62%	314 bp	98.30%	93.35%	18 197988 (92.44%)
RAL10[A #]	7,610,220	29.46%	382 bp	98.10%	92.47%	4 953 590 (65.09%)
RAL10[B #]	13,975,510	44.72%	333 bp	97.29%	91.47%	13 426 042 (96.07%)
RAL10[C #]	2,519,184	23.97%	333 bp	98.15%	92.70%	1 759 868 (69.86%)
Tonsil HD§	11,702,954	16.75%	333 bp	96.40%	88.95%	10 164 636 (86.86%)

€ The peaks size at 47 indicates adaptors, the second largest peak was in correct size range
For patient RAL10 three different biopises were sequenced in parallel
§ A BCR amplicon library from a healthy tissue tonsil was generated and used as control

Supplemental Table 7. Heavy chain VDJ-genes and CDR3 length for the five most frequent clonotypes.

Patient	Clonotype ID (%) §	CDR3 (aa)	VH gene	JH gene	Clear dominant clonotype identified
**RAL01**	ID22 (88.0%)	14	VH3-23	JH4	Yes
	ID57 (3.9%)	14	VH3-23	JH4	
	ID8 (1.03%)	18	VH3-23	JH6	
	ID81 (0.51%)	14	VH3-23	JH4	
**RAL03**	ID109 (77.5%)	16	VH3-21	JH6	Yes
	ID20 (12.0%)	8	VH3-73	JH4	
	ID102 (4.8%)	15	VH3-21	JH6	
	ID86 (1.5%)	15	VH3-21	JH4	
**RAL04**	ID124 (65.9%)	13	VH3-9	JH6	Yes
	ID78 (26.4%)	13	VH3-9	JH6	
	ID109 (1.03%)	15	VH3-9	JH4	
	ID223 (0.81%)	16	VH3-9	JH6	
**RAL05**	ID160 (48.7%)	63	VH1-2	JH6	No*
	ID218 (23.2%)	72	VH1-69	JH6	
	ID35 (11.3%)	45	VH3-7	JH4	
	ID177 (8.11%)	51	VH1-69	JH4	
**RAL07**	ID57 (72.2%)	18	VH3-15	JH6	Yes
	ID23 (20.7%)	17	VH4-31	JH4	
	ID3 (4.1%)	16	VH3-7	JH4	
	ID1 (0.67%)	14	VH3-7	JH4	
**RAL08**	ID83 (30.3%)	48	VH1-69	JH2	No*
	ID45 (25.2%)	39	VH1-18	JH4	
	ID65 (20.8%)	36	VH5-51	JH3	
	ID42 (9.25%)	48	VH3-9	JH4	
**RAL09**	ID9 (65.9%)	14	VH3-9	JH4	Yes
	ID13 (9.2%)	14	VH3-9	JH4	
	ID25 (9.1%)	18	VH3-9	JH6	
	ID29 (6.0%)	16	VH1-2	JH4	
**RAL10[A #]**	ID84 (99.1%)	20	VH1-2	JH4	Yes
	ID203 (0.27%)	22	VH1-2	JH6	
	ID137 (0.10%)	13	VH1-2	JH6	
	ID159 (0.09%)	16	VH1-2	JH6	
**RAL10[B #]**	ID214 (97.5%)	20	VH1-2	JH4	Yes
	ID218 (3.8%)	14	VH3-30	JH4	
	ID183 (0.28%)	14	VH3-30-3	JH4	
	ID206 (0.20%)	14	VH1-2	JH4	
**RAL10[C #]**	ID21 (97.5%)	20	VH1-2	JH4	Yes
	ID30 (0.008%)	16	VH1-2	JH2	
	ID7 (0.68%)	14	VH1-2	JH4	
	ID1 (0.30%)	13	VH1-2	JH4	

*Due to the uncertainty of dominating clonotypes these tissues were not continued with generation of monoclonal antibodies.
# For patient RAL10 three different biopises were sequenced in parallel which generated the same dominating clonotype.
§ Clonotype assignment was determined by Change-O: VH-JH gene usage, CDR3 length and CDR3 similarity.

Supplemental Table 8. Number of obtained productive filtered and annotated VH sequences per patient

Patient	RAL01	RAL03	RAL04	RAL05	RAL06	RAL07	RAL08	RAL09	RAL10€
**Total VH**									
VH unique sequences	2066	2287	3877	22013	777	3706	18574	173	40836
VH reads	59636	39403	77691	653955	7589	56084	683718	1483	1268666
**Dominating VH lineage§**									
Annotation	IGHV3-23 IGHJ4 CDR3 14aa	IGHV3-21 IGHJ6 CDR3 16aa	IGHV3-9 IGHJ6 CDR3 13aa	IGHV1-2 IGHJ6 CDR3 21aa	IGHV3-64 IGHJ6 CDR3 13aa	IGHV3-15 IGHJ6 CDR3 18aa	IGHV1-69 IGHJ2 CDR3 16aa	IGHV3-9 IGHJ4 CDR3 14aa	IGHV1-2 IGHJ4 CDR3 20aa
Sequences in lineage	1925	1314	2603	7402	226	2518	7261	124	38216
Reads in lineage	58323	30521	54332	318593	2681	40485	206935	1170	1253784
Reads in top 2 sequences# (% of all reads in lineage)	34528 (59%)	17824 (58%)	29313 (54%)	194899 (61%)	1909 (71%)	10990 (27%)	58374 (28%)	169 (14%)	309357 (25%)

Full length productive VDJ sequences after quality control filters and annotations
§ The most frequent lineage based on VH-JH gene usage and CDR3 length, this is a more inclusive clonotype definition than Chang-O annotation to include all variations.
€ Combined from three sequenced biopsies
The top two most frequence VH sequences were always identical except for one longer/shorter nucleotide at the start due to primer masking

Supplemental Table 9. Number of obtained productive filtered and annotated VL sequences per patient

Patient	RAL01	RAL03	RAL04	RAL05	RAL06	RAL07	RAL08	RAL09	RAL10 €
**Total VL**									
VL unique sequences	26734	105880	6322	N/A	51801	2834	N/A	61441	116419
VL reads	1827184	4501785	226998		2537022	61441		2685686	4431448
**Dominating VL lineage§**									
Annotation	IGKV2-28<br>IGKJ5<br>CDR3 9aa	IGLV1-51<br>IGLJ3<br>CDR3 11aa	IGLV3-1<br>IGLJ2<br>CDR3 9aa		IGKV1-12<br>IGKJ2<br>CDR3 9aa	IGKV4-1<br>IGKJ1<br>CDR3 9aa		IGKV1-39<br>IGKJ4<br>CDR3 8aa	IGLV2-18<br>IGLJ2<br>CDR3 12aa
Sequences in lineage	26464	88822	4622		31203	2203		20645	111335
Reads in lineage	1824395	4095319	201885		2231000	42971		1406226	4389803
Reads in the top 2 sequence# (% of all reads in lineage)	956168 (52%)	1573194 (38%)	92327 (46%)		1391265 (62%)	19772 (46%)		886688 (63%)	1172365 (27%)**

Full length productive VJ sequences after quality control filters and annotations
§ The most frequent lineage based on VL-JL gene usage and CDR3 length
Full length productive VJ sequences after quality control filters
€ Combined from three sequenced biopsies
The top 2 sequences are identical except a few nucleotides at the start or end due to primer masking
**top 4 sequences: 1658731 reads were identical with exception of the first nucleotides.

Supplemental Table 10. Controls mAbs used in polyreactivity assay

**Negative controls** [3, 6]	**Positive controls** [6]	**ACPA** [3, 4, 8-12] €	**anti-MDA/MAA** [1, 2]	**Lung** [10] #	**Gingiva** [13] #	**Bone Marrow** [8] #
1362:01E02	JB40	254:17D08	272:03G09	L201:12D01	GT01:01A11	254:16C05
1276:01G09	ED38	268:07G01	1276:01F04	L201:10D12	GT01:01B02	254:16F09
MGO53		254:C7X1604	1362:01H05	L201:02B06	GT01:01E10	254:17A03
		1003:10A01	146+01G07	L201:03F05	GT01:01E12	268:01E02
		1325:01B09	L201:10D01	L201:12A06	GT01:02A07	268:03D10
		1325:04C03	L201:11D02	L201:01E06	GT01:02B12	268:03H02
		1325:05C06	L204:03G05	L201:02D02	GT01:02C02	268:04A05
		1325:07E07		L201:10A08	GT01:02C05	268:04F04
		14CFCT2D09		L201:03G02	GT01:02E05	268:04F05
		14CFCT2H12		L201:10B09	GT01:02G04	268:05F05
		14CFCT3G09		L201:10F01	GT01:02H09	272:01A06
		62CFCT1E04		L201:10A04	GT01:03A01	272:02C08
		37CEPF1C11		L201:01B10	GT01:03A06	272:02G11
		37CEPF1C22		L201:11G11	GT01:03C01	272:03E05
		37CEPF1C71		L201:12A09	GT01:03C06	273:02D06
		37CEPF1C93		L201:12D11	GT01:03D04	273:02E01
		37CEPF2C05		L201:12H06	GT01:03D11	273:03E01
		37CEPT02C04		L205:05F04	GT01:03D12	273:03E06
		37CEPT1G09		L205:02A08	GT01:03E02	273:03H09
		37CEPF1C40		L206:01F08	GT01:03E06	274:02C04
		37CEPT1C08		L206:02A11	GT01:03G06	274:02D11
		BVCA1		L206:02B06	GT01:03G11	274:03B06
		L201:10D07		L206:01A12	GT01:03H01	274:03C04
		L201:11C11		L206:01B08	GT01:03H07	274:03D03
		L204:01A01		L206:02A07	GT01:03H11	274:03G07
		L204:01D07		L206:03D07	GT01:03H12	274:1bA06
		L204:05E10		L202:01C07	GT01:04A03	274:1bF03
		L205:09H06		L202:01G08	GT01:04A08	274:1bH07
		A4802:3286		L202:02C02	GT01:04B06	274:1bH08
				L202:02G10	GT01:04B10	254:C5X466
				L202:03A08	GT01:04B11	272:05G10
				L202:03G12	GT01:04C03	254:C5_No282
				L202:04E12	GT01:04D08	254:C7_No1276
				L202:04F12	GT01:04E09	
				L202:04H02	GT01:04G04	
				L204:06D02	GT01:04G05	

**Negative controls** [3, 6]	**Positive controls** [6]	**ACPA** [3, 4, 8-12] €	**anti-MDA/MAA** [1, 2]	**Lung** [10] #	**Gingiva** [13] #	**Bone Marrow** [8] #
				L204:06C01	GT01:04H03	
				L204:05B10	GT01:04H11	
				L204:03H12	GT01:05B03	
				L204:12H02	GT01:05B03	
				L204:12D12	GT01:05B04	
				L204:01F06	GT01:05B08	
				L207:01A02	GT01:05D04	
				L213:03F09	GT01:05D05	
				L204:05H08	GT01:05E11	
				L204:12C05	GT01:05H10	
				L211:03D11	GT06:01C01	
				L202:05H03	GT06:01C05	
				L204:02D01	GT06:01G01	
				L213:05G07	GT06:02B07	
				L213:07F12	GT06:02F06	
				L208:01A05	GT06:03A05	
				L211:04B06	GT06:03B03	
				L213:05E04	GT06:04A07	
				L213:05C08	GT06:04B03	
				L202:05H06	GT06:04F08	
				L213:03G07	GT06:04H09	
				L209:01B02		
				L202:04F11		
				L213:04D06		
				L204:05B07		
				L204:05B08		
				L202:06H02		
				L213:08D04		
				L204:01E02		
				L213:04D05		
				L201:02H10		
				L204:03G10		
				L202:05D08		
				L204:01A02		
				L204:05B01		
				L204:01B11		
				L213:02C01		
				L208:01A03		

**Negative controls** [3, 6]	**Positive controls** [6]	**ACPA** [3, 4, 8-12] €	**anti-MDA/MAA** [1, 2]	**Lung** [10] #	**Gingiva** [13] #	**Bone Marrow** [8] #
				L204:01D07		
				L202:01A09		
				L202:03A03		
				L202:06F11		
				L202:03B10		
				L202:02B05		
				L205:01D09		
				L204:06E12		
				L204:03F09		
				L206:01G01		
				L202:01H11		
				L207:01G02		
				L201:10F05		
				L207:07E03		
				L202:02G02		
				L201:10H06		
				L205:01F10		
				L205:09H06		
				L205:01C07		
				L211:03A08		
				L202:03G09		
				L207:07C02		
				L207:07A08		
				L206:02C11		
				L205:01B10		
				L204:03B12		
				L207:07C10		
				L207:08D04		
				L201:12D03		
				L201:01F10		
				L204:06H05		
				L201:12H03		
				L206:03E09		
				L202:01B11		
				L213:02F06		

€ All ACPA clones are CCP2 positive and positive for different cit-peptide reactivities [14].
Clones derived from lung (bronchoalveolar lavage fluid), gingiva tissue or bone marrow B cells/plasma cells without specific CCP2 or MDA/MAA reactivity.
All clones were produced as IgG1 using the same expression system (Expi293), purified by protein G chromatography and subjected to SDS-PAGE, ELISA and array analysis.

Supplemental acknowledgement, collaborators in the RA lymphoma group:

Johan Askling (Division of Clinical Epidemiology, Department of Medicine Solna, Karolinska Institutet, Stockholm, Sweden), Rose-Marie Amini (Department of Immunology, Genetics and Pathology, Uppsala University, Uppsala, Sweden), Gunilla Enblad (Department of Immunology, Genetics and Pathology, Cancer Immunotherapy, and Cancer Precision Medicine, Uppsala University, Uppsala, Sweden; Department of Oncology, Uppsala University Hospital, Uppsala, Sweden), Karin Ekström Smedby (Division of Clinical Epidemiology, Department of Medicine Solna, Karolinska Institutet, Stockholm, Sweden; Department of Hematology, Karolinska University Hospital, Stockholm, Sweden), Eva Kimby (Department of Hematology, Karolinska Institutet, Stockholm, Sweden), Lars Klareskog (Division of Rheumatology, Department of Medicine Solna, Karolinska Institutet, Stockholm, Sweden), Richard Rosenquist Brandell (Department of Molecular Medicine and Surgery Solna, Karolinska Institutet, Stockholm, Sweden), Christer Sundström (Department of Immunology, Genetics and Pathology, Uppsala University, Uppsala, Sweden), Eva Baecklund (Department of Medical Sciences, Rheumatology, Uppsala University, Uppsala, Sweden), Carin Backlin (Biology Education Centre, Uppsala University, Uppsala, Sweden: previously Department of Medical Sciences, Rheumatology, Uppsala University, Uppsala, Sweden), Alina Johansson (Department of Medical Sciences, Rheumatology, Uppsala University, Uppsala, Sweden), Charlott Mörth (Department of Oncology, Västerås Hospital, Västerås, Sweden; previously Mälarsjukhuset, Eskilstuna, Sweden), Max Flogegård (Department of Internal Medicine, Hematology, Falun General Hospital, Falun, Sweden), Tomas Husmark (Department of Internal Medicine, Rheumatology, Falun General Hospital, Falun, Sweden), Jörgen Lysholm (Department of Internal Medicine, Rheumatology, Falun General Hospital, Falun, Sweden), Annika Othzen Jerner (Department of Internal Medicine, Hematology, Gävle Hospital, Gävle, Sweden), Helena Gustafsson (Department of Internal Medicine, Hematology, Gävle Hospital, Gävle, Sweden), Tomas Weitoft (Department of Research and Development, Uppsala University/Region Gävleborg, Gävle, Sweden), Sven Tegmark (Department of Internal Medicine, Rheumatology, Gävle Hospital, Gävle, Sweden; Praktikertjänst, Gävle, Sweden), Karin Hallén (Department of Oncology, Karlstad Hospital, Karlstad, Sweden), Csaba Balaspiri (Department of Rheumatology, Karlstad Hospital, Karlstad, Sweden), Ingemar Lagerlöf (Division of Drug Research, Department of Medical and Health Sciences, Linköping University, Linköping, Sweden; previously University Hospital Linköping, Oncology, Linköping, Sweden), Per Eriksson (Länssjukhuset Ryhov, Jönköping, Sweden; previously University Hospital Linköping, Rheumatology, Linköping, Sweden), Christopher Sjöwall (Department of Biomedical and Clinical Sciences, Linköping University, Linköping, Sweden), Lena Brandefors (Department of Internal Medicine, Hematology, Sunderbyn Hospital, Luleå, Sweden), Maria Klosinska Linder (Department of Internal Medicine, Rheumatology, Sunderby Hospital, Luleå, Sweden), Katarina Wänkkö (Department of Internal Medicine, Rheumatology, Sunderby Hospital, Luleå, Sweden), Thomas Relander (Department of Clinical Sciences Lund, Section for Oncology and Pathology, Lund University, Lund, Sweden and Department of Oncology, Skane University Hospital, Lund, Sweden), Mats Jerkeman (Department of Clinical Sciences Lund, Section for Oncology and Pathology, Lund University, Lund, Sweden; Department of Oncology, Skane University Hospital, Lund, Sweden), Mats Ehinger (Department of Clinical Sciences, Pathology, Skane University Hospital, Lund University, Lund, Sweden), Elisabet Lindqvist (Section of Rheumatology, Department of Clinical Sciences Lund, Lund

University; Skane University Hospital, Lund, Sweden), Elke Theander (Department of Rheumatology, Skane University Hospital Malmö, Lund University, Lund, Sweden), Peter Olsson Department of Rheumatology, Skane University Hospital Malmö, Lund University, Lund, Sweden), Thomas Mandl (Department of Rheumatology, Skane University Hospital Malmö, Lund University, Lund, Sweden), Elisabeth Szekely (Department of Clinical Sciences Lund, Lund University, Sweden; Department of Oncology, Skane University Hospital Lund/Malmö, Sweden), Karin Hellgren (Clinical Epidemiology Division, Department of Medicine Solna, Karolinska Institutet, Stockholm, Sweden), Sara Harrysson (Department of Hematology, Cancer Theme, Karolinska University Hospital, Stockholm, Sweden), Stefan Norin (Oncopeptides AB, Stockholm, Sweden; previously Karolinska University Hospital Huddinge, Hematology, Stockholm, Sweden, Aase Hensvold (Department of Medicine, Rheumatology, Karolinska Universitetssjukhuset Solna, Stockholm, Sweden), Karin Papworth (Department of Oncology, Umeå University Hospital, Umeå, Sweden), Gerd-Marie Alenius (Department of Public Health and Clinical Medicine, Rheumatology, Umeå University, Umeå, Sweden), Hans Hagberg (Department of Immunology, Genetics and Pathology, Uppsala University, Uppsala, Sweden; Department of Oncology Uppsala University Hospital, Uppsala, Sweden), Mattias Mattsson (Department of Immunology, Genetics and Pathology, Uppsala University, Uppsala, Sweden; Department of Hematology, Uppsala University Hospital, Uppsala Sweden), Andrzej Piwowar (Department of Oncology, Västmanlands Hospital, Västerås, Sweden), Milad Rizk (Department of Rheumatology, Västmanlands Hospital Västerås, Sweden), Irena Mahmoodi (Department of Rheumatology, Västmanlands Hospital Västerås, Sweden), Urban Jerlström (Department of Oncology, Örebro University Hospital, Örebro, Sweden), Per Salomonsson (Department of Rheumatology, Örebro University Hospital, Örebro, Sweden)

REFERENCES

1. Sahlstrom P, Joshua V, Valkovskaia V*, et al.* Autoreactive B cells against malondialdehyde-induced protein cross-links are present in the joint, lung, and bone marrow of rheumatoid arthritis patients. *J Biol Chem* 2023; **299**: 105320.
2. Gronwall C, Amara K, Hardt U*, et al.* Autoreactivity to malondialdehyde-modifications in rheumatoid arthritis is linked to disease activity and synovial pathogenesis. *J Autoimmun* 2017; **84**: 29-45.
3. Lloyd KA, Steen J, Amara K*, et al.* Variable domain N-linked glycosylation and negative surface charge are key features of monoclonal ACPA: Implications for B-cell selection. *Eur J Immunol* 2018; **48(6)**: 1030-1045.
4. Steen J, Forsstrom B, Sahlstrom P*, et al.* Recognition of Amino Acid Motifs, Rather Than Specific Proteins, by Human Plasma Cell-Derived Monoclonal Antibodies to Posttranslationally Modified Proteins in Rheumatoid Arthritis. *Arthritis Rheumatol* 2019; **71**: 196-209.
5. Lloyd KA, Wigerblad G, Sahlström P*, et al.* Differential ACPA Binding to Nuclear Antigens Reveals a PAD-Independent Pathway and a Distinct Subset of Acetylation Cross-Reactive Autoantibodies in Rheumatoid Arthritis. *Front Immunol* 2019; **9**: 3033.
6. Wardemann H, Yurasov S, Schaefer A, Young JW, Meffre E, Nussenzweig MC. Predominant autoantibody production by early human B cell precursors. *Science* 2003; **301**: 1374-1377.
7. Huang W, Euler E, Lloyd KA*, et al.* Variable Fab domain N-glycosylation patterns in the B cell receptor repertoires of healthy individuals and patients with rheumatoid arthritis. *J Immunol* 2026; 215(6):vkag113
8. Hensvold A, Horuluoglu B, Sahlstrom P*, et al.* The human bone marrow plasma cell compartment in rheumatoid arthritis - Clonal relationships and anti-citrulline autoantibody producing cells. *J Autoimmun* 2023; **136**: 103022.
9. Titcombe PJ, Wigerblade G, Sippl N*, et al.* Pathogenic citrulline-multispecific B cell receptor clades in rheumatoid arthritis. *Arthritis Rheumatol* 2018; **70(12)**: 1933-1945.
10. Joshua V, Loberg Haarhaus M, Hensvold A*, et al.* Rheumatoid Arthritis-Specific Autoimmunity in the Lung Before and at the Onset of Disease. *Arthritis Rheumatol* 2023.
11. Hardt U, Carlberg K, Af Klint E*, et al.* Integrated single cell and spatial transcriptomics reveal autoreactive differentiated B cells in joints of early rheumatoid arthritis. *Sci Rep* 2022; **12**: 11876.
12. Germar K, Fehres CM, Scherer HU*, et al.* Generation and Characterization of Anti-Citrullinated Protein Antibody-Producing B Cell Clones From Rheumatoid Arthritis Patients. *Arthritis Rheumatol* 2019; **71**: 340-350.
13. Sherina N, de Vries C, Kharlamova N*, et al.* Antibodies to a Citrullinated Porphyromonas gingivalis Epitope Are Increased in Early Rheumatoid Arthritis, and Can Be Produced by Gingival Tissue B Cells: Implications for a Bacterial Origin in RA Etiology. *Front Immunol* 2022; **13**: 804822.
14. Mathsson Alm L, Westerlind H, Gehring I*, et al.* Recognition of Glycine Versus Nonglycine Citrulline Motifs Dictating the HLA Class II Association of Anticitrullinated Protein Antibodies: Insights From Autoantibody Profiling of 6,900 Scandinavian Patients With Rheumatoid Arthritis. *Arthritis Rheumatol* 2025; **77**: 1179-1193.
